# Cellular Characteristics and Protein Signatures of Human Adipose Tissues from Donors With or Without Advanced Coronary Artery Disease

**DOI:** 10.3390/biomedicines12112453

**Published:** 2024-10-25

**Authors:** Caitlin P. S. Ellis, Benjamin W. Tero, Christian M. Potts, Kimberly T. Malka, Xuehui Yang, Joshua Hamilton, Calvin Vary, Andre Khalil, Lucy Liaw

**Affiliations:** 1 MaineHealth Institute for Research, Scarborough, ME 04074, USA; caitlin.stieber@maine.edu (C.P.S.E.); benjamin.tero@mainehealth.org (B.W.T.); christian.potts@mainehealth.org (C.M.P.); kimberly.malka@mainehealth.org (K.T.M.); xuehui.yang@mainehealth.org (X.Y.); calvin.vary@mainehealth.org (C.V.); 2 Graduate School of Biomedical Science and Engineering, University of Maine, Orono, ME 04469, USA; joshua.hamilton@maine.edu (J.H.); andre.khalil@maine.edu (A.K.); 3 CompuMAINE Lab, Department of Chemical and Biomedical Engineering, University of Maine, Orono, ME 04469, USA

**Keywords:** human adipocyte, perivascular adipose tissue, coronary artery disease, proteomics, metabolism

## Abstract

**Background/Objectives**: Perivascular adipose tissue (PVAT) exerts a paracrine effect on blood vessels and our objective was to understand PVAT molecular signatures related to cardiovascular disease. **Methods**: We studied two groups: those undergoing mitral valve repair/replacement (VR, n = 16) and coronary artery bypass graft (CABG, n = 38). VR donors did not have coronary artery disease, whereas CABG donors had advanced coronary artery disease. Clinical and tissue pathologies and proteomics from adipose tissue were assessed. **Results**: Donors undergoing VR had a lower body mass index (*p* = 0.01), HbA1C (*p* = 0.0023), and incidence of diabetes (*p* = 0.022) compared to CABG. VR donors were overall healthier, with higher cardiac function compared to CABG donors, based on ejection fraction. Although adipose histopathology between groups was not markedly different, PVAT had smaller and more adipocytes compared to subcutaneous adipose tissues. These differences were validated by whole specimen automated morphological analysis, and anisotropy analysis showed small (2.8–7.5 μm) and large (22.8–64.4 μm) scale differences between perivascular and subcutaneous adipose tissue from CABG donors, and small scale changes (2.8–7.5 μm) between perivascular and subcutaneous adipose tissue from VR donors. Distinct protein signatures in PVAT and subcutaneous adipose tissue include those involved in secretion, exosomes and vesicles, insulin resistance, and adipocyte identity. Comparing PVAT and subcutaneous adipose tissue from CABG donors, there were 82 significantly different proteins identified with log fold change ≥ 0.3 or ≤−0.3 (*p* < 0.05). Using this threshold, there were 36 differences when comparing PVAT and subcutaneous adipose tissue from VR donors, 58 differences when comparing PVAT from CABG or VR donors, and 55 when comparing subcutaneous adipose tissue from CABG vs. VR donors. **Conclusions**: Routine histopathology cannot differentiate between PVAT from donors with or without coronary artery disease, but multiscale anisotropy analysis discriminated between these populations. Our mass spectrometry analysis identified a cohort of proteins that distinguish between adipose depots, and are also associated with the presence or absence of coronary artery disease.

## 1. Introduction

Cardiovascular disease (CVD) is the leading cause of death globally [1]. Obesity, diabetes mellitus, and metabolic disease increase the risk of cardiovascular disease [2]. These diseases present a significant strain on the medical system. Adipose tissue, which is comprised of immune cells, nerves, fibroblasts, and vessels, plays a key systemic role in these diseases due to its function as an endocrine and metabolic tissue [3]. We are focused on perivascular adipose tissue (PVAT) within the vascular microenvironment, as paracrine signaling regulates vascular function, and local changes can lead to systemic impacts.

PVAT is contiguous with the blood vessel adventitia and contributes cells and adipokines to the vessel wall [4,5]. In healthy individuals, PVAT secretes factors that decrease smooth muscle cell proliferation and increase relaxation of the smooth muscle, leading to normal lumen diameter and proper blood flow [6]. In the case of metabolic diseases such as obesity and diabetes, PVAT is expanded and altered secretory profiles lead to increased proliferation and contraction of the underlying smooth muscle, leading to a decreased lumen diameter and restricted blood flow [7,8]. Increased blood pressure is a risk factor for other cardiovascular outcomes, such as myocardial infarctions, strokes, and death [9]. We are interested in characterizing PVAT from a variety of donor populations in an attempt to understand and identify the specific factors that lead to the phenotypic change of PVAT.

There are three molecularly and functionally distinct types of adipose tissue—white, brown, and beige. White adipose tissue (WAT) primarily functions as lipid storage, with some endocrine effects [10]. WAT exists adjacent to the skin as subcutaneous (SubQ) fat, in the abdomen as visceral adipose tissue, and in the bone marrow. A recent study has described molecular features of white adipose tissues in mice and humans [11]. The primary function of brown adipose tissue (BAT) is heat production through non-shivering thermogenesis [12]. BAT is found in the interscapular region and the kidneys and expresses thermogenic markers such as UCP1 and GRP75. Activation of BAT has long been a research focus due to its capacity as a glucose sink and its role in insulin sensitivity [13]. In mice, it has been shown that adipose tissues have more brown adipocytes in response to injury, cold, and ß-adrenergic signaling, a process known as beiging [14]. Although morphologically, human PVAT appears like WAT, we are interested in the concept that human PVAT may have thermogenic qualities and can be stimulated to improve cardiovascular function or suppress disease. Indeed, some human adipose tissue, including human epicardial tissue, has been reported to have a beige or brown adipocyte profile [15,16], and we have reported similar evidence for human PVAT [17,18].

Our study was designed to test the hypothesis that human aortic PVAT associated with advanced coronary artery disease will be molecularly distinct from aortic PVAT from donors without coronary artery disease. Our objective was to define unique proteomic profiles in different adipose depots from the unique donor types studied. Our prediction was that PVAT from donors without atherosclerosis undergoing VR would represent more of a homeostatic phenotype, whereas PVAT from donors in the CABG group would reflect changes associated with metabolic dysfunction correlating with their vascular pathology.

Much of what is known about PVAT was defined in mice and other rodent models [4]. However, PVAT research in humans is rapidly growing. It has been shown that increased PVAT mass corresponds to increased atherosclerosis [19]. With regard to coronary artery disease, adipose tissue overlying the heart (peri-coronary) as well as PVAT adjacent to the aorta may impact coronary disease, in part due to secretion of pro-inflammatory cytokines [20]. Other related pathologies of coronary arteries, including coronary artery spasm has also been linked to PVAT inflammatory state [21]. Additionally, Shi et al. [22] characterized PVAT, subcutaneous, and epicardial adipose tissue in donors from the same cardiovascular disease groups presented here, undergoing VR or CABG surgeries. However, our populations differ demographically, leading to differences in experimental results. Specifically, Shi et al. [22] found no difference in body mass index between their groups and found increased fibrosis in PVAT compared to subcutaneous adipose tissue. We found that CABG donors have a significantly higher body mass index than VR donors and have found no differences in fibrotic tissue between disease and tissue types. Lastly, we have expanded upon this previous work through proteomics techniques to identify unique targets that distinguish between donor types and adipose tissue locations, and investigated the effect of diabetes in these tissues.

## 2. Materials and Methods

The overall experimental workflow is shown below:

 
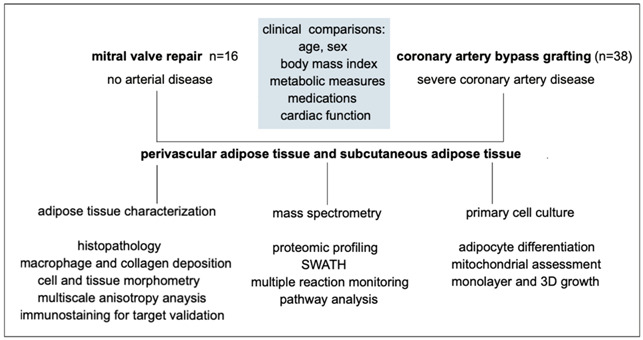


**Sample collection**. This work was approved by IRB# 1295440-3 at the MaineHealth Institute for Research. Donors were recruited from two groups undergoing open thoracic surgeries, either coronary artery bypass graft (CABG, n = 38) or mitral valve repair/replacement (VR, n = 16) surgeries. The groups were distinguished by the severity of coronary artery disease, where CABG donors had more atherosclerotic vessels than VR donors (Table 1). Our VR donors did not have diagnosed coronary artery disease, which was distinct from donors requiring CABG surgery due to advanced coronary artery disease. Because of this distinction, the VR group was considered the control group, and our experimental CABG group donors all had coronary artery disease. We documented written consent from tissue donors after sharing information about the research project and the request for collection of tissue that would otherwise be discarded. All samples were deidentified, and research staff had no access to the clinical information, which was maintained by the surgeon. Human perivascular adipose tissue (PVAT) and subcutaneous adipose tissue (SubQ) were collected from donors undergoing coronary artery bypass graft (CABG, n = 38) and mitral valve repair (VR, n = 16) surgeries. SubQ tissue was taken from the incision site and PVAT was collected from ascending aortae. Tissue processing was started within 4 h after collection. Because of the limitation of sample size available, we performed downstream analyses with a representative subset of samples as indicated in figure legends. 

**Tissue processing**. Tissue was processed, as described by Scott et al. [23], and was divided for histology, immunostaining, proteomics, and preadipocyte isolation. The amount of tissue was variable depending on initial sample size, and the order of prioritization was histology/immunostaining, preadipocyte isolation, SWATH, and multiple reaction monitoring proteomics. Samples were processed for paraffin embedding or frozen embedding. For the former, samples were fixed overnight in 10% formalin, washed three times in PBS, and stored in 70% ethanol at 4 °C until processing. Tissue dehydration and paraffin embedding were performed in our Histopathology and Microscopy core facility. Tissue was fixed in 10% formalin overnight, washed three times in PBS, and placed in 70% EtOH at 4 °C. Samples were processed for paraffin embedding and sectioning. Sections were stained with hematoxylin and eosin (H&E), Masson’s trichrome stain, imaged at 200x magnification, and analyzed with FIJI 2.9.0 H&E sections were analyzed using the AdipoSoft 1.6 extension [24]. Immunostaining was analyzed with color deconvolution using the H-dab and Masson’s trichrome features in FIJI.

For frozen sections, tissues were fixed in 10% formalin, and fixed tissue samples were incubated in 15% sucrose for 4 to 6 h, and then in 30% sucrose overnight at 4 °C. Samples were embedded in O.C.T compound (SAKURA Finetek, Torrance, CA, USA, cat# 4583) on dry ice, then subjected for cryosection at 7 μm thickness.

For immunofluorescence staining of frozen sections, slides were baked at 60 °C for 2 h, fixed with 10% formalin for 10 min, and then subjected to antigen retrieval using citrate buffer (10 mM citrate buffer containing 0.01% lithium dodecyl sulphate, pH = 6.6) for 5 min. Samples were incubated in 5% goat serum in PBS and 0.1% Triton-X for 1 h at room temperature. Co-immunofluorescence staining was performed by incubation with antibodies against UCP1 (R&D Biosystem, Minneapolis, MN, USA, cat# MAB6158, Lot CCNV0218031, 1:50 (10 μg/mL), Perilipin 1 (Cell Signaling Technology, Danvers, MA, USA, cat# 9349, lot 5, 1:100), Rab27a (Cell Signal Technology, cat# 69295, lot 2, (1.5 μg/mL), and Notch3 (Abcam, Waltham, MA, USA, cat# ab223426, lot 1059086-1 (4.5 μg/mL) at 4 °C overnight, followed by goat FITC-anti-mouse IgG (Vector Laboratories, Newark, CA, USA, cat# FI-2001, lot Y0118, 1:100), Alexa fluor 647-anti-mouse IgG (Invitrogen, Carlsbad, CA, USA, cat# A32728, 1:100), Alexa fluor 647-anti-Rabbit IgG (Molecular Probes, cat# A21244, lot 99E2-1, 1:100), and Alexa fluor 488-anti-Rabbit IgG (Invitrogen, cat# A11008, 1:100. Normal mouse IgG1 or rabbit IgG (Cell Signaling Technology, cat# 5415, lot 10, or cat# 3900, lot 50, respectively), used accordingly for control staining. Nuclei were stained with DAPI. Images were captured using a Leica SP8 confocal microscope.

Samples for preadipocyte isolation were washed in HBSS with 2x antibiotic/antimycotic solution prior to processing. Samples for proteomics were stored frozen at −80 °C prior to processing.

**Histological staining and analysis.** Tissue sections were stained with H&E or Masson’s trichrome stain. Slides were placed in hematoxylin (Richard-Allan Scientific, Kalamazoo, MI, USA, cat# 7231) for 10 min at room temp and washed in tap water until clear. Slides were then placed in 5% acetic acid for 1 min and washed in tap water, followed by Bluing Agent (Richard-Allan cat# 7301) for 2 min and a tap water wash. Slides were placed in 95% EtOH for 1 min, stained in Eosin Y (Richard-Allan cat# 7111) for 2 min and rinsed in two 95% EtOH washes for 30 s each. Tissues were dehydrated in three changes of 100% EtOH, cleared, and mounted with synthetic resin (Permount, Electron Microscopy Sciences, Hatfield, PA, USA).

For Masson’s trichrome stain, 40 mL of Bouin’s fixative (Newcomer Supply, Middleton, WI, USA, cat# 1020A) was placed in a coplin jar and heated in the microwave for 30 s. Sections were placed in heated solution for 20 min, allowed to cool, and rinsed in running tap water for 10 min. Sections were stained in Weigert’s hematoxylin (Richard-Allan cat# 7231) for 10 min. Samples were washed in water for 10 min and stained in Biebrich’s scarlet-acid fuchsin (EKI, Joliet, IL, USA, cat# 26905) for 10 min. Samples were washed for 10 min and stained in phosphotungstic/phosphomolybdic (Sigma-Aldrich, St. Louis, MO, USA, cat# MKCT3045) solution for 10 min. Slides were placed in aniline blue solution (Newcomer, cat# 10073C) for 10 min, rinsed in tap water, and placed in 1% acetic acid for 3–5 min. Samples were washed in water for 1 min, 95% EtOH for 1 min, dehydrated, and mounted with synthetic resin.

H&E sections were imaged at 200x and analyzed using the AdipoSoft extension in FIJI. Ten fields of view per sample were imaged and analyzed for adipocyte size, number, and stromal area.

**Sliding Window and Anisotropy Analysis** (see also Appendix A). H&E sections were imaged on the Keyence BZ-X810 microscope at 20x magnification. Images were then loaded into the scikit-image (ver 0.22.0) library in Python (ver 3.10). Each whole slide image (WSI) underwent a color transformation into hue, saturation, and value from RGB. The saturation channel was made into a black and white binary mask using the Otsu automated thresholding method. This created a tissue mask, in which a bounding box divisible by 1024 was fit to contain the entire mask. The top left corner of the bounding box was used as the top left coordinate to begin the sliding window analysis. The sliding window consisted of 1024 × 1024 pixel subregions from the original RGB WSI and were slid across the image in 256-pixel x-coordinate increments until meeting the right edge of the bounding box. The y-coordinate window was then shifted down a 256-pixel increment and the process continued until reaching the bottom right coordinate of the bounding box.

Each subregion was first color transformed to hue, saturation, value, and the saturation channel used for segmentation of adipocytes. The segmentation began with a 3-pixel Gaussian blur into a Triangle threshold on the saturation channel from the color transformed subregion creating a binary foreground and background image. This resulted in stained material as background and unstained material as foreground, allowing for watershed segmentation of white adipose tissue. Any objects touching the border were removed.

The following thresholds, determined from manual inspections, were used to remove gaps in stromal tissue, vessels, ducts, or low-quality stained adipocytes from the segmentation thereby ensuring inclusion of only whole adipocytes. Any objects with an area larger than 3674 µm (9000 pixels) and smaller than 653.2 µm (1600 pixels) were removed. Objects with areas greater than 10,206 µm, 25,000 pixels, and were highly eccentric (>0.87) were removed as well as any object with a perimeter to area ratio of 0.14 or greater. After all these thresholds, if the area of the subregion was occupied by at least 30% segmented objects, every object’s x-centroid, y-centroid, eccentricity, area, perimeter, long-axis, and short-axis were saved into a comma-separated-value (CSV) file to calculate morphometrics. Saved objects whose centroids were within a 12.7 µm (31 pixel) radius of each other were randomly removed until only one remained preventing oversampling of the same adipocyte from the overlapping sliding window analysis. Morphological analysis for area, solidity, circularity, and eccentricity was performed. Subregions that passed all these requirements and had minimal (<20%) overlap with previously saved subregions were also saved as patches for anisotropy analysis.

A custom Python implementation of the 2D Wavelet Transform Modulus Maxima Anisotropy method was used to probe the organization of adipose tissue from these patches at a range of different size scales [25,26,27]. Critically important observations can be missed when using a computational image analysis tool that only considers a single scale (or a single frequency, if using a Fourier-based method) and the 2D WTMM Anisotropy method overcomes this by acting as a “mathematical microscope” through utilization of continuous wavelet transforms. The method was developed in the context of astrophysics and has since been ported to multiple biological contexts including collagen, soft-tissue in growth, nerves, and most recently, cancer [25,26,27,28,29]. This is the first application of the technique on H&E-stained adipose tissue. The adipocyte morphometrics and anisotropy measurements were calculated and tested using Wilcoxon ranked sum between all four groups (VR PVAT vs. VR SubQ, CABG PVAT vs. CABG SubQ, VR PVAT vs. CABG PVAT, and VR PVAT vs. VR SubQ). 

**Immunostaining.** Sections were deparaffinized and rehydrated. Tissue was outlined with a PapPen, dried, and rinsed in tap water. Antigen retrieval was done as specified for each antibody by the data sheet. The slides were rinsed twice in PBS and twice in TBS-T (Tris buffered saline, pH = 7.6, with 0.05% Tween). Slides were blocked in 2% normal serum blocking buffer (secondary host serum, 1% BSA) for 30 min at room temperature and washed twice in TBS-T. Slides were then blocked with the A/B kit (Vector, Cat.# SP2001) and washed in TBS-T twice. Slides were incubated with primary antibody (MAC1, Abcam, cat.#ab133357) overnight at 4 °C. Slides were washed four times with TBS-T and incubated with secondary antibody diluted in PBS. Slides were washed three times in TBS-T, blocked with endogenous peroxidase with 3% H_2_O_2_ in PBS, and washed in TBS-T twice. Samples were incubated with ABC amplification reagent (Vector, Cat.# PK6100) and washed in TBST four times. Slides were incubated in working 3,3′-Diaminobenzidine (Sigma, F8764-5G), washed in tap water, dehydrated, cleared, and mounted.

**Stromal vascular fraction isolation.** The stromal vascular fraction was isolated according to the procedure in Scott et al. 2019 [23]. Briefly, adipose tissues were washed in HBSS with 2x antibiotic/antimycotic solution, digested in collagenase (Millipore, Burlington, MA, USA, cat# 11088815001), spun down at 500× *g* for 8 min, and plated on a gelatin coated plate, typically, a 12 well plate. Cells were incubated in a humidified chamber at 37 °C and 5% CO_2_. Cells were fed every four days with growth media (DMEM/F12, 10% FBS, 1x antibiotic/antimycotic) and split at ~95% confluence (1:2, and passage number recorded). Propagated cells were cryopreserved and utilized for downstream characterization. 

**Lipid Detection.** Fixed cells were brought to room temperature and incubated with 1:200 dilution (concentration is proprietary, I called Invitrogen) of LipidTOX 488 (Invitrogen, Cat.# H34475) and 2.5 μg/μL concentration of DAPI (Invitrogen, Cat.# D1306) for 1 h at room temperature. The cells were washed three times in PBS and stored at 4 °C. Cells were brought to room temperature, placed in fresh PBS and imaged on the LI-Cor (Lincoln, NE, USA) Odyssey-M Imager and Keyence (Itasca, IL, USA) BZ-X810 microscope using the 488 cube filter and UV filter. Three full wells per sample were imaged at 20x magnification. Images were quantified using FIJI for intensity of LipidTOX stain per nuclei.

**Data visualization and statistical analysis.** Anisotropy was visualized using a Python library. Statistical analysis was done with a Wilcox or Mann–Whitney *t*-test. GraphPad Prism (version 10) was used for all other student t-tests and two-way ANOVA analysis, as well as a graphic visualization of quantifiable data.

**Mass spectrometry and proteomics analysis.** Samples were prepared as described by Rappsilber et al. [30]. Briefly, samples cooled to room temperature, alkylated, and protein precipitated. Pellets were washed in ethanol, centrifuged, and suspended. Digested proteins evaporated and were subjected to solid phase extraction on C18 resin. Purified peptides were eluted into autosampler vials and resuspended in a volume of sample load solvent (5% formic acid (Optima grade, ThermoFisher Scientific, Waltham, MA, USA) and 4% acetonitrile (LC-MS-grade, Honeywell, Morris Plains, NJ, USA) to yield ~1 µg/µL peptides.

**Chromatography/Mass spectrometry.** Tandem mass spectrometric analysis was performed on an Eksigent NanoLC 425 nano-UPLC System (Sciex, Framingham, MA, USA) in direct-injection mode with a 5 µL sample loop. Fractionation was performed on a reverse-phase nano HPLC column (Acclaim PepMap 100 C18, 75 µm × 150 mm, 3 µm particle, 120 Å pore) held at 45 °C with a flow rate of 350 nL/min. 

Analysis was performed in positive ion mode on a TripleToF 5600 quadrupole time-of-flight (QToF) mass spectrometer (Sciex, Framingham, MA, USA). The column eluate was directed to a silica capillary emitter (SilicaTip, 20 µm ID, New Objective, Littleton, MA, USA) maintained at 2500 V. A parent ion scan was acquired, followed by MS/MS analyses of the 50 most-intense ions detected in the parent ion scan. SWATH MS/MS windows of variable widths, 100 in total, were generated using a variable-window calculator (Sciex). Fragmentation conditions were optimized for ions of a 2+ charge state. SWATH detection parameters were set to a mass range of *m*/*z* = 100–1500 with accumulation times of 50 msec in the high-sensitivity mode. 

**Proteomics Data analysis.** Protein identification was performed using Protein Pilot software (Sciex, Version 5.0.2) running the Paragon algorithm. Data were searched against a human proteome database on the Uniprot website. Searches were performed with cysteines modified (iodoacetamide). A target false discovery rate of 0.05 and a thorough ID search effort was selected. A minimum of 95% confidence was used as a threshold for peptide identification. Relative quantification was performed using the SWATH processing microApp in the Sciex PeakView ver 2.2 software. Peak groups were extracted with a 99% peptide confidence threshold and 1% peptide FDR limit. SWATH chromatograms were extracted in 10-min windows with fragment ion mass tolerance set to 75 ppm. Resulting protein quantitative peak areas were further analyzed using MarkerView software (Version 1.3.1, Sciex LLC, Framingham, MA, USA) to compare relative quantities of all detected proteins between samples. Statistical analyses, including *t*-tests and principal component analyses, were completed for data sets using Sciex MarkerView ver 1.3.1 software. Significantly different proteins were determined via *t*-test (*p* < 0.05). In STRING db, a minimum required interaction score of 0.9 was used (highest confidence) and GO terms were sorted based on *p*-value and FDR. Only proteins that were statistically different in levels between comparison groups are listed in the Supplemental Tables. Raw data are in the PRIDE repository under reference number 1-20240423-213918-2242764, accession number PXD051676.

**Multiple Reaction Monitoring (MRM) Proteomics.** Peptides were prepared as above and subjected to LC/MS and MS/MS fragments (Q1 and Q3 transmission, respectively) (Sciex (Framingham, MA, USA) 6500+ triple quadrupole mass spectrometer). Candidate peptides for MRM detection and quantitation were selected either from primary data acquired by SWATH acquisition using a high resolution quadrupole TripleToF 6600+ system and Eksigent (Dublin, CA, USA) Ekspert 425 liquid chromatograph as described above, or by using the human MRM library on the SRMAtlas utility [31]. For SRMAtlas selection of candidate Sciex QTRAP SRM, Q1 and Q3 ions were chosen from the Human SRMAtlas 2024-01 build, with Target instrument set to QTRAP5500, and y- and b-ions without modifications. Peptides were resolved using a custom hand-made C18 reverse-phase column with a 1.7 µL per minute flow rate and a 10–80% ACN/0.1% FA gradient. MRM was conducted in positive ion mode recommended by SRMAtlas. Results were sorted based on a *p*-value of <0.05, a log fold change of ≤ −0.3 or ≥0.3, and visualized using R version 4.3.2.

**Immunoblotting.** Tissue and cell samples were digested in a RIPA buffer with a protease inhibitor, spun down at 12,800× *g* for 10 min, and the supernatant was saved. The protein concentration was assessed using the DC protein assay (cat.#) and samples were diluted with a Laemmli buffer. Samples were run on 12% gels at 120 volts until the protein front reached the bottom, transferred using a semidry transfer at 1.5 amps for 30 min, and were blocked in 5% milk in TBS-T for 1 h at room temperature. Samples were placed in a primary antibody (insert table) overnight up to 72 h, washed, and in secondary at 1:1000 for 2 h at room temperature, before digital detection with forte HRP substrate.

**Preadipocyte 3D Spheroid Culture and Differentiation.** Cells were dissociated using 0.25% trypsin/EDTA (Gibco 65200-056) for 5 minutes, neutralized with growth media and centrifuged at 500× *g* for 8 min. An amount of 10,000 cells were plated into each well of a 96-well round bottom spheroid plate (NunclonSphera 174925, BioFloat F202003) by diluting to 200,000 cells/mL and adding 50 uL to each well. An additional 50 uL of growth media was added to each well. Cells were incubated for three days to allow spheroid formation. On day three, media was removed around spheres using manual pipetting, 100 uL of induction media (Table 1) was added to cells for differentiation, and growth media was added to control cells. Cells were incubated for three more days, at which point the differentiated cells were changed into maintenance media (Table 1). Regular media changes occurred every Monday and Friday for the duration of the experiment. At ten days, one batch of differentiated cells with controls were moved into an Eppendorf tube, fixed in 10% Formalin, and washed in PBS, then stored at 4 °C. The rest of the cells were differentiated for a total of eighteen days, before being moved into an Eppendorf tube, fixed with 10% Formalin, washed in PBS, and stored at 4 °C.

**Oil Red O Staining of Spheroid Adipocyte Cultures.** Spheres were removed from storage Eppendorf tubes into new tubes for staining and were blocked using a solution of 0.2% Triton X-100, 3% BSA, and PBS for 40 min at room temperature. Blocking solution was then removed and the cells were stained in a solution of 2.5μg/μL DAPI (Invitrogen, Cat. # D1306) and a 1:40 dilution of Phalloidin 488 (Invitrogen A12379) in blocking solution for 1 h at room temperature. Spheres were then washed three times in PBS before being stained in a solution of six parts of 50 mg/mL Oil Red O (Sigma Aldrich #O0625) in 6 mL water and four parts water for 2 h at room temperature. Spheres were then washed in a solution of six parts isopropanol and four parts water quickly, before subsequent washing in water until red color was removed. Stained spheres were mounted in water in a glass bottom petri dish and imaged using a Leica SP8 confocal microscope using a 20x magnification objective.

## 3. Results

### 3.1. Distinct Clinical and Physiological Features of Patients with Coronary Artery Disease

Adult patients undergoing target surgeries were consented for sample collection; multiple concurrent surgeries led to exclusion. Adipose tissue was taken from two populations, those undergoing mitral valve repair (VR) or coronary artery bypass graft (CABG) surgery. Table 2 summarizes their demographic information (see also Supplemental Table S1). The median age was not different between surgical groups, 66 and 70.5 for VR and CABG, respectively. The proportion of donors on lipid lowering (and anti-hypertension drugs) also did not significantly differ between groups. However, 33.3% of VR patients were on lipid lowering drugs compared to 81.1% of the VR group. Additionally, 56.3% of VR donors are on anti-hypertension drugs, compared to 76.3% of CABG donors. Body mass index (25.26 vs. 29.96), HbA1c (5.45 vs. 5.90), incidence of diabetes (6.25% vs. 36.8%), and donors on anti-diabetic medication (6.25% vs. 37.8%) were all lower in the VR donors compared to CABG donors. CABG donors had more vessels on average that had atherosclerotic legions compared to VR donors. CABG patients also had a lower ejection fraction. Overall, these data indicate that the VR donor group were healthier compared to the CABG donor group.

### 3.2. Morphological and Protein Expression Differences in PVAT and SubQ Point to Changes in Adipose Functionality

**Figure 1:**
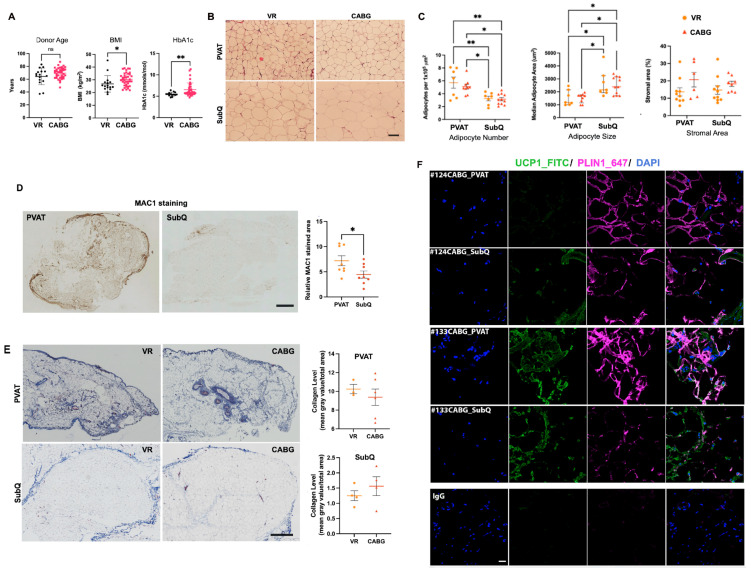
**A comparison of PVAT and SubQ between VR and CABG donors.** (**A**) While there was no significant difference in age of donors from VR vs. CABG groups, the CABG group had higher body mass indices (BMIs) and circulating HbA1c levels. (**B**) Representative images of adipose tissue sections after hematoxylin/eosin staining for PVAT or subcutaneous adipose (SubQ) from donor groups. Scale bar = 50 μm. (**C**) Morphometric analysis was performed to quantify adipocyte number, size, and stromal area. Adipocytes were smaller in PVAT compared to SubQ regardless of disease incidence. VR PVAT n = 7, VR SubQ n = 7, CABG PVAT n = 9, CABG SubQ n = 11. (**D**) Sections were immunostained to detect MAC1 to quantify macrophages in adipose tissue in CABG PVAT (n = 8) and SubQ (n = 8). Representative images are shown, and quantification indicated higher macrophage presence in CABG PVAT compared to CABG SubQ. Scale bar = 750 µm. (**E**) Sections were stained with Masson’s trichrome stain to identify collagen. There was no detectable difference between CABG and VR PVAT or VR and CABG SubQ. Scale bar = 500 µm. VR PVAT n = 3, CABG PVAT n = 6, VR SubQ n = 4, CABG SubQ n = 4. (**F**) Sections from human adipose tissues from CABG donors were immunostained to detect UCP1 (green) and perilipin1 (PLIN1, pink). Shown is the merge in the right column. The bottom row represents negative control staining with non-immune IgG. Scale bar = 20 μm. * *p* < 0.01, ** *p* < 0.001.

Given the demographic differences between donor groups, we were interested in assessing differences in their adipose tissue. We examined the histopathology of the tissue by analyzing adipose morphology, tissue fibrosis, and macrophage infiltration (Figure 1A–C). Adipocyte size, number, and stromal area were quantified using AdipoSoft in FIJI. In all three measures, there were no significant differences between conditions (VR vs. CABG). However, within each disease condition, PVAT had more adipocytes that were smaller compared to those in subcutaneous adipose tissue. In the VR group, PVAT had a median of 5.72 adipocytes per 1 × 10^5^ μm^2^ compared to 3.26 adipocytes per 1 × 10^5^ μm^2^ in VR SubQ. In the CABG group, PVAT had a median of 5.10 adipocytes per 1 × 10^5^ μm^2^ compared to 3.16 adipocytes per 1 × 10^5^ μm^2^ in subcutaneous adipose tissue.

Stroma is the area between adipocytes in the tissue. There were no significant differences between groups in the stromal area and the difference in adipocyte number was fully accounted for by the difference in size. The stromal area was 15.5%, 16.9%, 15.4%, and 16.9% for VR PVAT, CABG PVAT, VR SubQ, and CABG SubQ, respectively. We then analyzed tissue macrophages by immunostaining with MAC1, which is a macrophage marker and indicative of inflammation. There were no significant differences between the donor groups, but there was a significantly higher area of MAC1 stain in the CABG PVAT samples compared to CABG SubQ adipose tissue (Figure 1D). The connective tissue of all four groups was assessed using Masson’s trichrome stain (Figure 1E). There were no significant differences in collagen presence between disease types in PVAT or SubQ. PVAT VR had a ratio of mean stained area/total area of 10.24 vs. 9.38 in CABG VR, and VR SubQ had a mean stained area of 1.25 compared to 1.57 in subcutaneous adipose tissue from CABG donors. 

We further localized adipocyte markers to human adipose tissue, focusing on perilipin1 (PLIN1), a lipid droplet membrane protein, and UCP1, a marker of thermogenic adipose tissue. Using immunofluorescence, we found that all samples had significant PLIN1 staining in the tissue, as expected. Although more variable, we also did detect UCP1 protein in samples from PVAT and SubQ adipose tissue from our donors. Although we had hypothesized that UCP1 might be more representative in PVAT compared to SubQ, or VR versus CABG tissue, we found variability between subjects with no clear difference between adipose depot and donor type.

### 3.3. Sliding Window Analysis of Adipocyte Morphology Shows PVAT Adipocytes Have a Smaller Area than SubQ Adipocytes

**Figure 2:**
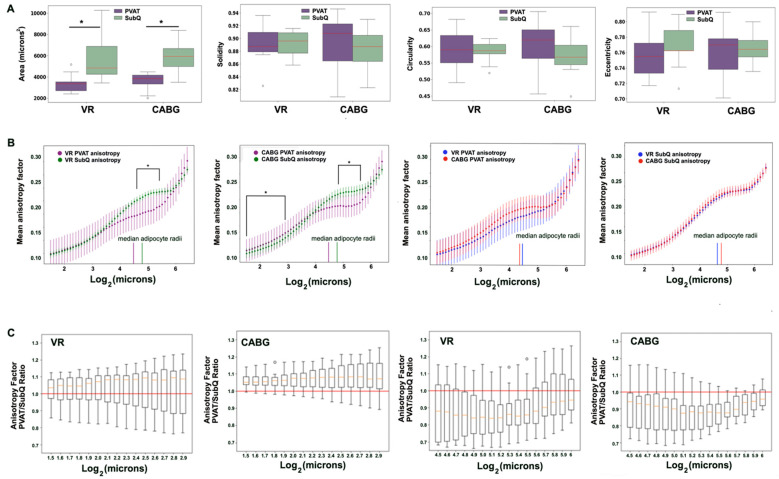
**A morphometric and anisotropy analysis of tissues with sliding window segmentation validate FIJI analysis.** (**A**) A morphometric analysis of patient adipocytes from the sliding window segmentation analysis where patient median values from all their adipocytes are plotted. Quantified are area, solidity, circularity, and eccentricity. Significance, shown as asterisks, were calculated between patient tissue subtypes and across surgery subtypes using an unpaired Wilcoxon ranked sum test with an α of 0.05. (**B**) Anisotropy Factor plots of patient mean adipose tissue anisotropy factors and standard deviation across 50 wavelet size scales of VR PVAT vs. SubQ, CABG PVAT vs. SubQ, VR PVAT vs. CABG PVAT, and VR SubQ vs. CABG SubQ. All automated sliding window H&E 1024 × 1024 subregions that passed manual inspection of adipocyte content were used to calculate each of the patients’ mean anisotropy factors. Significance, shown as asterisks, were calculated at each wavelet scale using an unpaired Wilcoxon ranked sum test with an α of 0.05. The median adipocyte radii in microns of each category are shown as vertical bars with colors corresponding to the legend. (**C**) Each donor’s mean PVAT Anisotropy Factor subtracted from their mean SubQ Anisotropy Factor and divided by their sum is plotted as a boxplot over ranges of statistically significant size scales shown in panel B. Small scale anisotropy ratios are plotted for VR and CABG from 2.8 microns to 7.5 microns. Large scale anisotropy ratios are plotted for VR and CABG from 22.8 microns to 64.4 microns. A horizontal line at 1.0 is shown as red to indicate the location of where the median anisotropy factor from a donor’s PVAT tissue is equivalent to the anisotropy factor from their SubQ tissue. * *p* < 0.01.

Histological tissue slides were analyzed using a sliding window analysis to assess adipocyte area, circularity, eccentricity, and solidity. As with the manual subregion selection FIJI analysis, only the adipocyte area was significantly different (VR area *p* = 0.008, CABG area *p* = 6.5 × 10^−5^, Figure 2A). Within each disease type, PVAT had smaller adipocytes by area than adipocytes from subcutaneous adipose tissue. This corroborates the manually selected subregions data and confirms that there were no significant differences in adipocyte size, circularity, eccentricity, or solidity between VR and CABG derived tissues.

### 3.4. The Multiscale Anisotropy Analysis Shows Differences at Both Small (2.8 to 7.5 μm) and Large (22.8 to 64.4 μm) Scales

The anisotropy factor was analyzed to compare the adipose depots (perivascular versus subcutaneous) within VR donors or CABG donors, or the same adipose tissue depot between donor types (VR versus CABG). The anisotropy factor measures structural organization across multiple size scales and can be used to assess changes in tissue and cell organization. Scales smaller than the size of an adipocyte (small) indicate changes in the membrane while scales larger than the adipocytes (large) indicate organizational changes on the cell and tissue level. VR PVAT vs. VR SubQ comparison showed only large scale changes, indicating differences in tissue organization, whereas CABG PVAT vs. CABG SubQ had both small and large scale changes (Figure 2B,C). These data suggest that in CABG patients, changes in the PVAT may represent single adipocyte membranes as well as overall organization. There were no significant differences in anisotropy factors between VR and CABG PVAT or VR and CABG SubQ. The patient-level normalized difference anisotropy plots (Figure 2C) indicated a greater variation at small scales between SubQ and PVAT tissue organization in VR patients when compared to CABG. Overall, this corroborates the manual subregion FIJI and automated sliding window data confirming that there were limited differences in tissue organization within adipose tissues between disease types.

### 3.5. Diabetes Does Not Significantly Affect Adipose Tissue Morphology in CABG Patients

**Figure 3:**
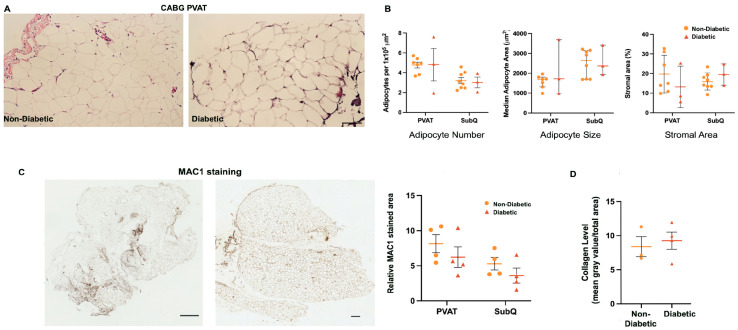
**The diabetes status associated with few changes in CABG PVAT and SubQ.** (**A**) Hematoxylin/eosin staining, and (**B**) morphometric comparisons of adipocyte size, number, and stromal area in CABG PVAT accounting for diabetes. Non-diabetic PVAT n = 7; diabetic PVAT n = 3; non-diabetic SubQ n = 8; diabetic SubQ n = 3. Scale bar 50 µM. (**C**) The MAC1 immunostaining in CABG and SubQ from donors with and without diabetes of stained area is shown in the graph on the right. Non-diabetic PVAT n = 4; diabetic PVAT n = 4; non-diabetic SubQ n = 4; diabetic SubQ n = 4. Scale bar 250 µm and 50 µm, respectively. Scale bar = 50 µm. (**D**) The quantification of tissue fibrosis using Masson’s trichrome Masson in non-diabetic and diabetic PVAT and SubQ. Non-diabetic PVAT n = 3; diabetic PVAT n = 4.

Given the high incidence of diabetes in the CABG cohort and the common comorbidity of diabetes and cardiovascular disease, we investigated whether adipose tissue was distinguishable between diabetic and non-diabetic donors (Figure 3). There were no significant differences in adipocyte size, number, or stromal area between PVAT and SubQ regardless of diabetes incidence (Figure 3A,B). PVAT from donors without diabetes averaged 1696 μm^2^, with 4.78 adipocytes per 1 × 10^5^ μm^2^, and 19.7% stromal area versus 1725 μm^2^, 4.81 adipocytes per 1 × 10^5^ μm^2^, and 13.19% stromal area in PVAT from donors with diabetes. SubQ from donors without diabetes averaged 2650 μm^2^, 3.21 adipocytes per 1 × 10^5^ μm^2^, and 15.96% stromal area versus 2368 μm^2^, 3.02 adipocytes per 1 × 10^5^ μm^2^, and 19.5% stromal area in SubQ from donors with diabetes. There was no difference in the MAC1 stained area (Figure 3C) or the collagen area between non-diabetic and diabetic patients (Figure 3D).

### 3.6. Proteins Involved in Adipogenesis, Insulin Resistance, and Secretion Are Differentially Expressed Between Donor Groups and Adipose Depots

**Figure 4:**
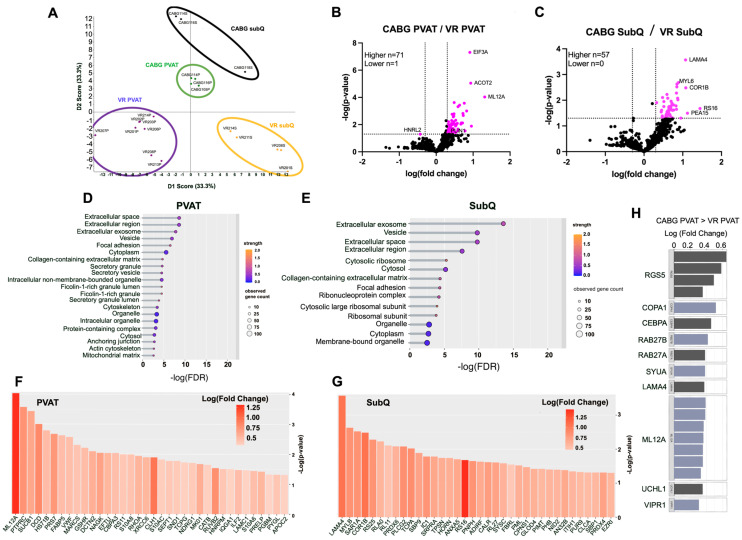
SWATH proteomics reveals differential protein expression in proteins involved in adipogenesis, secretion, and insulin resistance. VR 201 donor had shunt placed ~16 years prior to the VR surgery. (**A**) PCA plot showing differences in 4 SWATH groups: CABG PVAT (n = 4, green), VR PVAT (n = 6, purple), CABG SubQ (n = 3, black), and VR SubQ (n = 5, yellow). (**B**) Volcano plots highlight the proteins of interest that are expressed differently between CABG and VR PVAT. (**C**) Volcano plots highlight the proteins of interest that were significantly differently expressed (pink dots) between CABG and VR SubQ. (**D**) STRING analysis highlighted gene ontology cellular component terms in all differentially expressed proteins between the PVAT. Terms are classified by false discovery rate (FDR), strength (internal STRING measurement), and observed gene count. (**E**) STRING analysis highlighted GO cellular component terms in all differentially expressed proteins between the SubQ groups. Terms were classified by false discovery rate (FDR), strength, and observed gene count. (**F**) Proteins that are classified in gene ontology terms “extracellular space”, “extracellular region”, “extracellular exosome”, and “vesicle” for PVAT were classified by *p*-value and log fold change for CABG PVAT/VR PVAT. (**G**) Proteins that are classified in gene ontology terms “extracellular space”, “extracellular region”, “extracellular exosome”, and “vesicle” for PVAT, were classified by *p*-value and log fold change for CABG SubQ/VR SubQ. (**H**) MRM targeted proteomics showed log-fold change in proteins of interest that were higher in CABG than VR PVAT. Multiple peaks per peptide is indicative of multiple ions for that peptide.

Given the paracrine signaling function of PVAT, we were interested in differences between the patient groups on a protein scale. SWATH proteomic analysis was done using MS/MS (Figure 4). Principle component analysis (PCA) plots were generated to determine how similar the protein expression of each group was. Based on PCA, all four groups of interest segregate independently and have distinct protein expression (Figure 4A). Proteins of interest that are higher and lower in CABG PVAT/VR PVAT (Figure 4B) and CABG SubQ/VR SubQ (Figure 4C) are labeled in the volcano plots. Proteins involved in adipogenesis, insulin resistance, and secretion are higher in CABG PVAT. Of particular interest are PLIN1, MGLL, and SYUA which are all higher in CABG PVAT compared to VR PVAT. Additionally, PEA15 was higher in subcutaneous adipose tissue from CABG donors compared to VR donors. Perilipin (PLIN1) is a lipid coat protein. The higher the level of PLIN1, the more adipose that is present. PLIN1 being increased in CABG PVAT compared to VR PVAT fits with our hypothesis that PVAT in CABG patients is expanded and has more adipose.

The gene ontology (GO) of cellular component terms were ordered based on the false discovery rate (FDR), strength from STRING, and the observed gene count. Between CABG and VR PVAT, the terms with the highest FDR (indicative of most likely to be a true difference) were extracellular space, extracellular region, extracellular exosome, and vesicle (Figure 4D). Comparing subcutaneous adipose tissue from CABG and VR donors, the terms with the highest FDR included extracellular exosome, vesicle, extracellular space, and extracellular region (Figure 4E). GO terms for biological process and molecular function were also investigated using STRING, but no differences of note were observed. We then took the proteins classified under the GO terms “extracellular exosome”, “vesicle”, secretory granule”, and “secretory vesicle” and examined significance (*p*-value) and log-fold change for both PVAT (Figure 4F) and subcutaneous adipose tissue (Figure 4G) comparisons. In both heat bar graphs, the darker the color indicates a higher log fold change, while the length of the bar indicates significance. Proteins are sorted from most to least significantly different. Given the involvement of secretory proteins, as well as interest in validating the SWATH proteomics, we used multiple reaction monitoring to assess protein expression.

### 3.7. Targeted Proteomics for SWATH Validation

Multiple reaction monitoring proteomics were performed on CABG PVAT and SubQ, both non-diabetic and diabetic, and VR PVAT (Figure 4H). Proteins were prioritized based on their identification in the bulk mass spectrometry analyses. Multiple reaction monitoring is a way to select specific peptides of interest out of a library and search for them within a tissue sample [32]. Multiple peptides and multiple ions for each protein can be detected, which often leads to multiple peaks for a single protein, including some that may be upregulated and some downregulated for the same protein, or even the same peptide.

The first goal was to validate the first round of SWATH proteomics. To this end, PLIN1, COL25A1, MYO1B, SYUA, ML12A, and LAMA4 were tested because these proteins were differentially expressed in the first round (Figure 4H). Of note, ML12A was higher in CABG PVAT compared to VR PVAT, where in the original SWATH data it was lower. LAMA4 was detected as higher in in CABG PVAT when compared to VR PVAT. RS16 and PEA15 were not differentially expressed.

**Figure 5:**
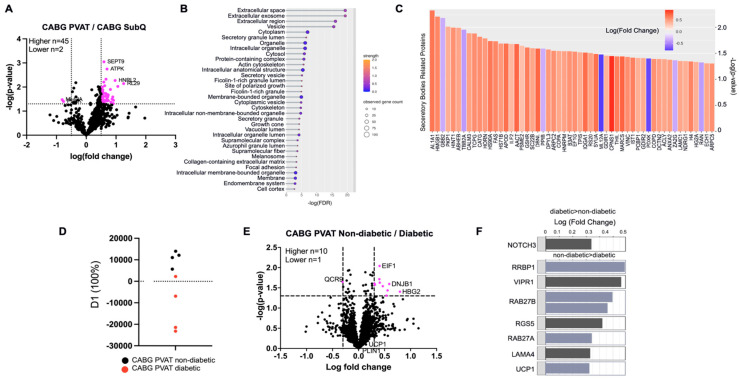
**SWATH proteomics of non-diabetic and diabetic CABG PVAT.** (**A**) From the first SWATH data set, the volcano plot shows significant protein expression differences (pink dots) between CABG PVAT and SubQ. In this data set, there was a mix of diabetic and non-diabetic samples. (**B**) STRING analysis of proteins differentially expressed between CABG PVAT and SubQ displaying enriched cellular component gene ontology terms. (**C**) Proteins that are classified in gene ontology terms “extracellular space”, “extracellular region”, “extracellular exosome”, and “vesicle” in PVAT/SubQ comparison were classified by *p*-value and log fold change for CABG PVAT/CABG SubQ. (**D**) PCA showed a clear distinction between non-diabetic and diabetic CABG PVAT. (**E**) Volcano plot identified ten proteins higher in non-diabetic than diabetic CABG PVAT. (**F**) Proteins significant in MRM proteomics, displayed by log fold change. Each bar represents an individual ion.

In addition to validating the SWATH proteomics, we investigated protein expression of proteins involved in secretion (RAB27A, RAB27B), innervation (PGP9.5), thermogenesis (GRP75 and UCP1), and pericyte markers (VIPR1, RGS5). Using multiple reaction monitoring (Figure 4H), we found that RAB27A and RAB27B are higher in CABG PVAT compared to VR PVAT. This indicates both an increase in secretion and adiposity, which may partially account for the increased PVAT in CABG patients. PGP9.5 (UCHL1) was higher in CABG PVAT than VR PVAT, suggesting that CABG PVAT is more highly innervated than VR PVAT. PGP9.5 was also higher in the perivascular versus subcutaneous adipose depot from CABG donors (Figure 5). There was no difference in thermogenic marker UCP1 or GRP75 expression in PVAT from CABG or VR donors. VIPR1 and RGS5 were both higher in PVAT from CABG donors compared to VR donors, indicating an increased presence of pericytes in these adipose depots. Pericytes are mural cells of capillaries and also regulate insulin secretion in B-islet cells [33].

### 3.8. Comparison of Adipose Depots Within CABG Donor Groups

The protein expression between the perivascular and subcutaneous adipose depots from CABG donors was compared using proteomics via MS/MS. A total of forty-five proteins were higher in PVAT and two were lower compared to subcutaneous adipose tissue from CABG donors. Proteins of interest are highlighted in Figure 5A. All significantly different proteins were then analyzed using STRING. Top hits included extracellular exosome, vesicle, and secretory granule. This suggested that secretion may be higher in CABG PVAT. Lastly, the proteins in the GO terms “extracellular exosome, vesicle, secretory granule, and secretory vesicle” were examined for log fold change differences (Figure 5C). Overall, most of the secretory proteins were more highly expressed in CABG PVAT, providing further evidence for differences in secretion.

### 3.9. Analysis of PVAT Protein Profiles of Diabetic vs. Non-Diabetic CABG Donors

Given the high presence of diabetic patients in the CABG cohort, as well as the common comorbidity of diabetes and cardiovascular disease, we investigated whether diabetes was associated with a unique protein signature. In this comparison, PVAT from CABG donors was separated into non-diabetic and diabetic patients (Figure 5). The PCA shows a difference in protein pattern between the non-diabetic and diabetic groups (Figure 5D). A total of ten proteins were higher in the non-diabetic PVAT than the diabetic PVAT and one was lower (Figure 5E). Thus, the proteomic differences between groups were driven by these eleven proteins identified that distinguished between PVAT from non-diabetic or diabetic donors. UCP1 and PLIN1 were detected in these samples but was not different between groups when analyzed by bulk proteomics. However, in the targeted proteomics (Figure 5F), UCP1 expression was higher in PVAT from diabetic donors. This is potentially indicative of a protective mechanism in the diabetic tissue leading to adipose browning.

SWATH proteomics were validated using multiple reaction monitoring (Figure 5F). We specifically tested QCR9 and HBG2 as they were the lowest and highest differentially expressed proteins based on log fold change in expression. In the multiple reaction monitoring between CABG PVAT from diabetic donors and CABG PVAT from non-diabetic donors, QCR9 was significantly different with a *p*-value of 0.035 and a non-significant log fold change of 0.22. This trend was consistent with the original SWATH proteomics showing lower QCR9 expression in non-diabetic PVAT. HBG2 was also not differentially expressed but given a *p*-value of 0.057 and a log fold change of −0.4; this indicates that HBG2 is lower in non-diabetic PVAT, which also paralleled the SWATH proteomics data. In addition to these changes, protein changes in secretory proteins (RAB27A and RAB27B), a thermogenic protein (UCP1), and pericyte markers (NOTCH3, VIPR1, and RGS5) were investigated. NOTCH3 was higher in diabetic PVAT, but all other proteins were more highly expressed in the non-diabetic PVAT.

### 3.10. Localization of RAB27A and NOTCH3 Within Human Adipose Tissue

**Figure 6:**
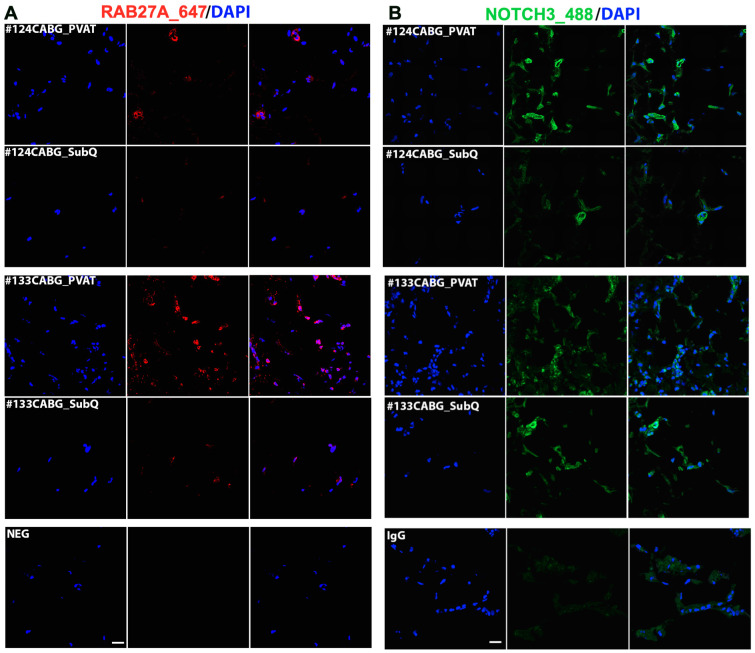
**The detection of RAB27A and NOTCH3 in human adipose tissue.** Antigens were selected for validation for the proteomics. (**A**) Shown are representative images of immunofluorescence staining in two CABG donor tissues, with matching PVAT or subcutaneous (SubQ) adipose tissue from each donor. The negative control (NEG) was a secondary antibody treatment only. Scale bar = 20 μm. (**B**) The same samples were analyzed for NOTCH3 protein localization. Non-immune IgG staining is shown in the bottom row. Scale bar = 20 μm.

Immunofluorescence was used to validate protein expression in adipose tissues (Figure 6). RAB27A and NOTCH3 were detected in both perivascular and subcutaneous depots from CABG donors. Because RAB27A is higher in CABG PVAT compared to VR PVAT (Figure 4H), and higher in diabetic versus non-diabetic CABG PVAT (Figure 5F), we localized it within the tissue. We consistently observed higher levels of RAB27A protein in PVAT compared to subcutaneous adipose from CABG donors, which validates our proteomics result. RAB27A was localized to puncta localized around the nucleus and is expected to be associated with cellular membranes in its trafficking and secretion functions. NOTCH3 was detected in a mural wall of blood vessels and also in cells not associated with vessels. These NOTCH3 cells are of interest, as recent evidence indicates that smooth muscle cells and pericytes populations may comprise a unique adipocyte progenitor population [18]. Further characterization of pericytes in CABG and VR adipose tissue is ongoing.

### 3.11. Differentiation of Human Preadipocytes Is Associated with Elevated Mitochondria

**Figure 7:**
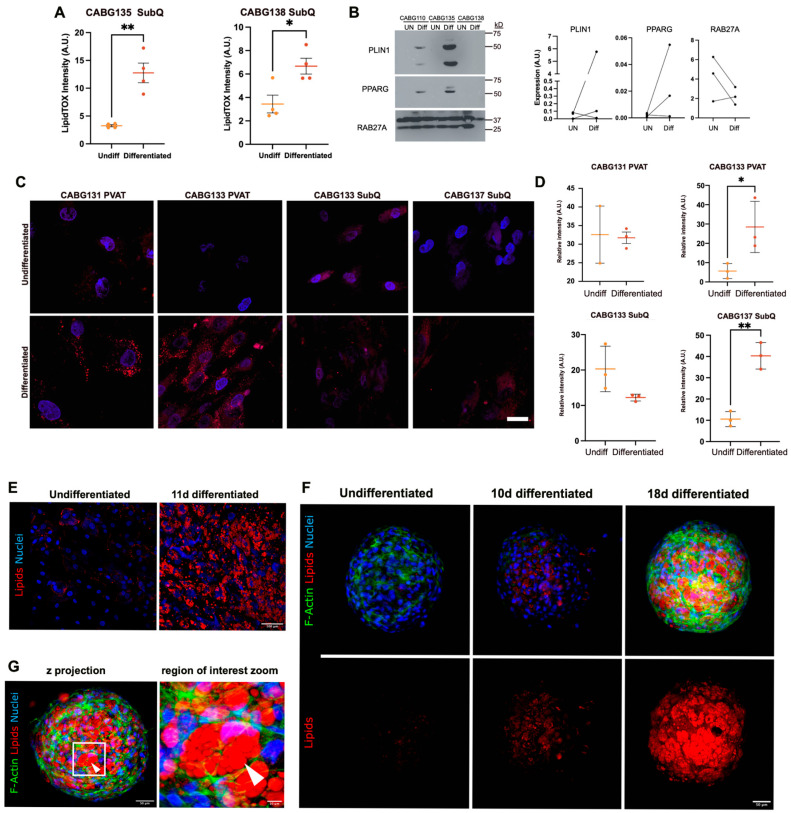
**The differentiation of human preadipocytes and impact on mitochondria**. Various preadipocyte populations from CABG donors were either left under standard growth conditions or differentiated as described for nine days. (**A**) LipidTOX was used to detect lipids based on fluorescence intensity. (**B**) Immunoblot of PLIN1 (62 kD), PPARG (57, 53 kD), and RAB27A (27 kD) following differentiation (Diff) compared to undifferentiated (UN) controls. Quantification of the immunoblots are shown in the graphs on the right. (**C**) Mitotracker was used to stain mitochondria as indicated (red) in undifferentiated or differentiated populations. Scale bar = 25 μm. (**D**) Quantification of the mitotracker stain from groups shown in (**C**). *p*-value * ≤ 0.05, ** *p*-value ≤ 0.01. (**E**) Human PVAT-derived preadipocytes were grown in monolayer culture and differentiated into adipocytes for eleven days. Nuclei were stained with DAPI (blue), and Oil Red O (red) was utilized to detect lipids. (**F**) Human preadipocytes were obtained from PVAT from donors undergoing CABG and grown in 3D spheroid culture as described. Cells were either left undifferentiated in a standard growth medium or differentiated for ten or eighteen days in an adipogenic differentiation medium. Nuclei were stained with DAPI (blue), Oil Red O (red) was utilized to detect lipids, and F-actin was visualized with phalloidin. (**G**) An eighteen-day differentiated spheroid is shown, with a higher magnification region of interest showing the lipid droplets.

In addition to an analysis of the whole adipose tissue, we isolated cells from the stromal vascular fraction that can be propagated and differentiated into mature lipid laden adipocytes. This model is important because we can manipulate the cells and study pathways of interest, which is not feasible in vivo. This experiment served two important purposes: (1) to confirm that human preadipocytes can be isolated and differentiated from our donor populations and (2) to confirm expression of protein targets of interest. Primary cultures of these human stromal vascular fraction-derived populations were differentiated using standard conditions. Because of variability in propagating individual human adipose tissue samples, and generally small pieces of PVAT obtained, we performed these experiments on cells derived from subcutaneous adipose tissue. Two independent isolates showed differentiation capacity (Figure 7A), based on cellular lipid accumulation quantified by LipidTOX. Expression of adipocyte markers PLIN1 and PPARG were assessed by immunoblot, and there was some variability in rat and extent of differentiation between donor populations (Figure 7B). RAB27A was also assessed. In two out of three primary cell populations, the RAB27A decreased upon differentiation, which has been shown previously [17]. Undifferentiated and differentiated adipocytes were assessed for mitochondrial presence and activity using MitoTracker (Figure 7C,D). The number of mitochondria increased upon differentiation. Additionally, fission was seen in the differentiated populations.

## 4. Discussion

**VR donors are metabolically healthier than CABG donors.** Samples were collected from both males and females, however the donors were primarily male, consistent with a higher proportion of males undergoing CABG procedures than females [34]. Globally, 30% of CABG patients are female [34,35], while in our cohort, the proportion of females was only 7.9%. Based on the national inpatient database from 2005–2009, between 60 and 65% of mitral valve replacements were performed on females [36,37], while only 31% of our VR cohort was female. Given that diabetes is a risk factor for atherosclerosis [38], it is expected that the patients with severe arterial disease (CABG) are more likely to be diabetic. Additionally, VR donors had a lower BMI than CABG patients and were less likely to be on anti-diabetic medication. Finally, cardiac functional analysis indicated that the VR population had a significantly higher ejection fraction compared to the CABG donors. Collectively, these data indicate the overall better health of VR patients compared to the CABG patients.

**Morphological analysis shows limited differences in adipose between donor groups.** The only significant differences we found in adipocyte number was in comparison between the perivascular and subcutaneous adipose depots. PVAT has more adipocytes, despite no significant differences in size or stromal area. However, adipocyte size appears to be trending larger in subcutaneous adipose tissue, which fits with the adipocyte size data. The stromal area was not different between groups. This was potentially due to the large size of the adipocytes, leaving a relatively low percentage of stromal area. However, we did find higher levels of MAC1-positive macrophages in perivascular versus subcutaneous adipose tissue from CABG donors, potentially representing a higher inflammatory state. A possible explanation for increased inflammation is the closer proximity of the PVAT to the site of atherosclerosis, as the adipose could be affected by inflammation within the vasculature. Previous studies have shown that increased adipose tissue fibrosis is correlated with increased adiposity and body mass index [39]. However, we detected no differences in tissue fibrosis detected between our donor populations or adipose depots. In our assessment of the association of adipose tissue morphology with diabetes, we did not detect any significant differences. The modest differences observed in the protein signatures based on diabetes status was consistent with this. We believe that the medical management of diabetes may account for the adipose tissue phenotypes being very similar at the morphological and molecular level.

**The level of tissue organization differs between PVAT and SubQ.** In CABG donors, both perivascular and subcutaneous adipose depots displayed small and large scale changes in anisotropy. Our team has been working on novel applications of computational multiscale approaches to enhance biophysical understanding of disease for biomedical applications. While this approach has been utilized to study human cancer [25], this is a unique application to human adipose tissue. The large scale changes can be interpreted as differences in cellular organization that are undetectable with other analyses. Because this analysis is performed on histological slides and represents primarily data on adipocyte plasma membranes, we are interested in the concept that the anisotropy measurement reflects differences in plasma membrane structure or function. An important function of adipose tissue is its endocrine function of secretion of paracrine signaling molecules. We are particularly interested in this secretory function of PVAT to underlying blood vessels.

**Proteomic analysis shows changes in proteins involved in adipogenesis, insulin resistance, and secretion.** Perilipin (PLIN1) is a lipid coat protein that is positively correlated with adipose tissue expansion. In PVAT, our observation that PLIN1 was higher in CABG compared to VR donors was consistent with our hypothesis that adipose tissue expansion is elevated in CABG donors, corresponding to their decreased metabolic health. However, we would also expect there to be an accompanying change in adipocyte size which was not seen. Some attention has focused on the capacity of clinical imaging to monitor adipose tissue expansion or inflammation with relation to metabolic disease or risk for cardiovascular disease, which may provide some context in combination with cellular and molecular studies. Another differentially regulated protein identified to be highest in PVAT from CABG donors was monoglyceride lipase (MGLL), which is involved in the endocannabinoid system and mediates signaling in the brain and peripheral tissues, including adipose [40]. Obesity is associated with higher levels of MGLL expression, and three genes associated with higher BMI are also associated with increased MGLL expression [40,41]. Our result fits what is known about MGLL expression, because CABG patients in our study were more likely to be overweight and obese than VR donors. The effect of MGLL is tissue dependent and has an effect on insulin sensing and glucose tolerance. Given what is known about MGLL function, it could be a potentially interesting target for the controlling of adipogenesis and has implications to insulin sensitivity in diabetes. Another protein that may be controlling insulin sensitivity and was higher in PVAT from CABG donors was alpha synuclein (SYUA). SYUA is often associated with the formation of Lewy bodies in Parkinson’s disease (PD), but has recently been shown to be involved in the regulation of insulin sensitivity as well as lipid storage in both mice and humans [42,43]. Low levels of SYUA in humans are associated with insulin resistance, while an increase in SYUA is associated with increased adiposity, in addition to increased insulin sensitivity. Given that SYUA was more highly expressed in CABG PVAT, this is another piece of evidence supporting increased adiposity in CABG PVAT. However, the accompanying implication of increased insulin sensitivity, given the CABG donors higher propensity for insulin resistance, seems to counteract this point.

We identified phosphoprotein enriched in astrocytes 15 (PEA15) in human subcutaneous adipose tissue at higher levels in CABG versus VR donors. This is of interest because PEA15 is involved in type 2 diabetes and is a recently discovered regulator of adipose tissue expansion [44]. In mice, PEA15 null mice have improvements in whole body insulin sensitivity, lower liver weight, and decreased serum triglyceride. As far as we are aware, this work is the first time PEA15 expression has been examined in humans. Our proteomics results suggest that PEA15 has a similar role in insulin sensitivity and adipose tissue expansion seen in mice.

The SWATH proteomics data suggested that secretion may be changed between disease states in both perivascular and subcutaneous adipose tissues. RAB27A and RAB27A are RAS family proteins responsible for trafficking vesicles to the membrane. Boucher et al. showed that human preadipocyte differentiation was inhibited with suppression of RAB27A [17]. Differential expression of these RAB proteins in human adipose depots may reflect changes in protein trafficking and secretion. Interestingly, mutation in *RAB27A* in humans can lead to type II Griscelli syndrome, which is reflected in impaired protein trafficking resulting in hypopigmentation and changes in immune responses [45]. In mice, mutation in *Rab27a* leads to impaired exocytosis of insulin from pancreatic beta cells in response to glucose [46], and we recently discovered that *Rab27a* global null mice have cardiovascular dysfunction in an age- and sex-dependent manner [47]. These results suggest that these pathways may be conserved between mice and humans in regulation of cardiometabolic physiology.

Since we were interested in increased differentiation and adiposity, we are also interested in innervation changes, as it has been previously shown that chemically ablating adipose tissue innervation leads to increased fat pad mass in subcutaneous white adipose [48]. In ob/ob mouse models, there was decreased innervation of both the subcutaneous white adipose depot and the interscapular brown adipose tissue [49]. This study also found that in human white adipose tissue, lower levels of the neural marker PGP9.5 were found in those with a higher body mass index. In our study, the highest level of PGP9.5 was found in the PVAT from CABG donors, who also had the highest body mass index.

Thermogenic markers have long been used as an indicator of the relative health of brown and beige adipose tissue. Human PVAT morphologically resembles white adipose tissue, although it produces UCP1 and other thermogenic markers [17], allowing for use of these markers to determine the degree of beiging in adipose. We hypothesized that PVAT from VR versus CABG donors would be metabolically healthier and thus have higher thermogenic marker expression. However, we found significant and relatively equivalent levels of these proteins in PVAT regardless of donor type, suggesting again that human PVAT maintains thermogenic phenotype.

The increased expression of proteins related to secretion and thermogenesis in non- diabetic PVAT fits with the hypothesis that non-diabetic PVAT has more brown adipocytes than diabetic PVAT. However, we showed that this relationship does not always hold true, and likely represents variations between individuals. Thus, more investigation is needed to accurately determine the effect of these proteins in the adipose.

**Overall Conclusions.** Human coronary artery disease is highly prevalent, and we utilized the clinical study populations described to assess whether PVAT was molecularly distinguishable in patients with coronary artery atherosclerosis versus those with other types of cardiac pathologies. Our major conclusions are: (1) patients without coronary artery disease have a healthier metabolic and clinical profile, including lower BMI and circulating HbA1c levels, significantly lower diabetes prevalence, and significantly higher cardiac function, as measured by ejection fraction. There was no difference in the age of the donor populations. (2) Adipocytes from human subcutaneous depots are larger than those in PVAT regardless of disease state, and these adipocytes can be distinguished using multiscale anisotropy analysis of histological sections. The significant changes in PVAT and subcutaneous anisotropy factors from CABG donors suggest changes at the single cell membrane level and tissue organization. (3) Major proteomic differences in the adipose depots between donor groups included functional categories such as exosomes, vesicles, extracellular space, and secretory granules/vesicles, supporting changes in the secretory activity of the adipocytes. (4) Human preadipocyte differentiation into lipid-containing adipocytes in vitro is associated with increased mitochondria, and differentiation occurs in monolayer culture as well as 3D spheroid culture. These models can be used further for cell-based assays of human adipocyte function.

**Study Limitations**. Our study provides novel information about adipose tissue from two types of donors with distinct vascular pathologies, with samples from either subcutaneous or perivascular adipose depots. Starting to define donor-specific and depot-specific molecular markers was the goal of this study. We validated the clinical and molecular differences in PVAT between the donor populations and presented some case studies of the protein targets of interest and the cellular behavior of human primary preadipocyte populations. The limitations of the study are the small amount of PVAT that could be carried on for downstream analysis; not all applications were possible with all samples. We had different levels of success in deriving primary cell cultures from the donor tissues, and this may represent variability in cellular growth rates or survival capacity. In addition, although the donors were clearly distinguished by the CABG group having several coronary artery diseases, and the VR group not having any coronary artery diseases upon surgery, some donors had additional cardiac-related surgeries at the same time as the primary procedure; 34% of the CABG donors and 87.5% of the VR donors had other concomitant cardiac surgeries. Thus, it is possible that other preexisting conditions or pathologies affected the PVAT from these individuals. Despite these limitations, the ability to study molecular and cellular behavior of primary human adipose tissue is a benefit and will increase translational capacity of research findings.

## Figures and Tables

**Table 1:** The components of adipocyte differentiation media.

Factor	Concentration	Media Type
DMEM/F-12 (50/50) (Corning, Corning, NY, USA, cat# 10-0920-CV)	1x	Induction/maintenance
Heat inactivated-FBS (R&D systems, cat# S11550H)	1x	Induction/maintenance
Antibiotic and antimycotic (Corning, cat# 30-0040-CL)	1x	Induction/maintenance
Insulin (Sigma, cat# 91077C-250 mg)	170 nM	Induction/maintenance
T3 (Sigma, cat# TG397)	2 nM	Induction/maintenance
Rosiglitazone (Sigma, cat# R2408)	1 μM	Induction/maintenance
IBMX (Sigma, cat# R2408)	0.5 mM	Induction
Dexamethasone (Sigma, cat# D4902)	5 μM	Induction
Indomethacin (Sigma, cat# I8280)	125 μM	Induction
TGF-β RI Kinase Inhibitor VI (Cell Signaling, cat# 14775)	5 μM	Induction/maintenance
L-Ascorbic acid 2-phosphate sesquimagnesium salt hydrate (Sigma, cat# A8960)	50 μg/ml	Induction/maintenance

**Table 2:** **A comparison of donor populations**. Lipid-lowering medications include low to high intensity statins such as atorvastatin, rosuvastatin, and simvistatin. Anti-hypertensive medications include linisopril, amlodipine, metoprolol, digoxin, hydrochlorothiazide, torsemide, warfarin, nifedipine, diltiazem, torsemide, acetaminophen, and amiodarone. Additional data are in Supplemental Table S1. ns = not significant.

	VR	CABG	*p* Value
Total sample (female)	16 (5)	38 (3)	
Median age (variance)	66 (14.5)	70.5 (13.5)	ns
Median body mass index (variance)	25.3 (4.9)	30 (8.32)	0.01
Median HbA1c (variance)	5.45 (0.48)	5.9 (1.45)	0.0023
Median # atherosclerotic vessels (variance)	0 (1.1)	3 (2.1)	2.24 × 10^−7^
Donors with diabetes	1 (6.3%)	14 (36.8%)	0.022
Donors on anti-diabetic medication	5 (33.3%)	30 (81.1%)	0.022
Donors on lipid lowering drugs	5 (33.3%)	30 (81.1%)	ns
Number on anti-hypertension drugs	9 (56.3%)	29 (76.3%)	ns
Overall ejection fraction			0.043
Ejection fraction > 55%	13 (81.2%)	22 (59.5%)	
Ejection fraction 50–55%	3 (18.8%)	2 (5.4%)	
Ejection fraction 40–50%	0	8 (24.3)	
Ejection fraction 30–40%	0	1 (2.7%)	
Ejection fraction < 30%	0	3 (8.1%)	

**Figure A1:**
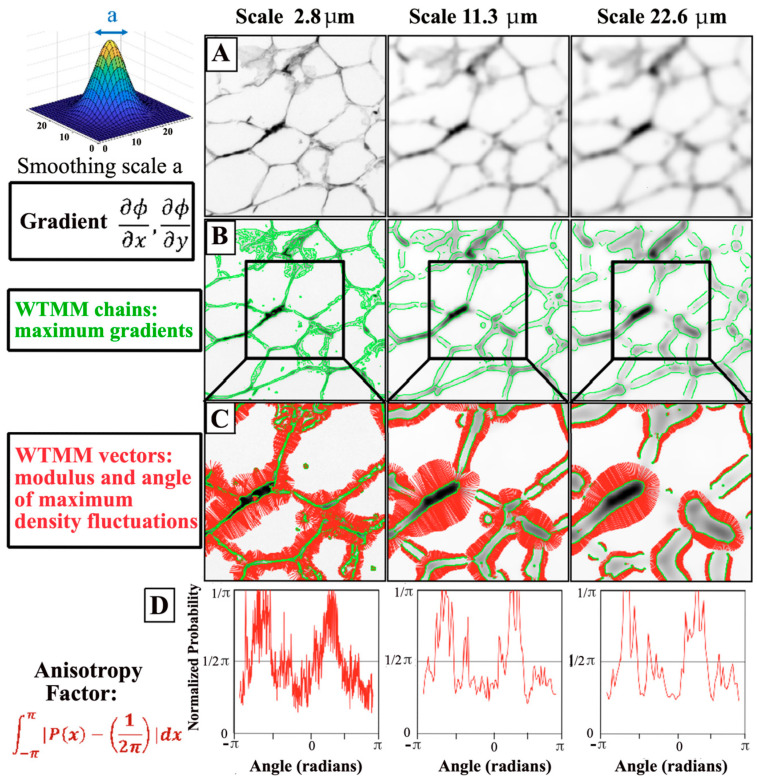
(**A**) A Gaussian wavelet (Φ) convolved with an adipocyte image taken from a PVAT sample at three representative wavelet size scales. (**B**) Maxima chains representing local maximal gradients, i.e., the wavelet-transform modulus maxima (WTMM), at three representative wavelet scales shown in green. (**C**) Magnified inserts from the white outlined area in panel B at three representative wavelet size scales to identify WTMM vectors (red) showing the magnitude and direction of local gradients of the maximum image density fluctuations. (**D**) Probability density functions (PDF), shown in red, of three representative wavelet scales generated from angles of local gradient vectors with the theoretical isotropic 1/2π line shown in black. The anisotropy factor (see equation in red, where P(X) is the PDF at one scale) is obtained by integrating the difference between each PDF and the theoretical 1/2π line.

## Data Availability

Proteomics raw data are in the PRIDE repository under reference number 1-20240423-213918-2242764, accession number PXD051676. All other raw data are available upon request.

## Supplementary Materials

The following supporting information can be downloaded at: https://www.mdpi.com/article/10.3390/biomedicines12112453/s1, Supplemental proteomics methodology, Supplemental Table S1 (full demographic information), and Supplemental Tables S2–S6, with lists of proteomic differentially regulated proteins between comparison groups.
