# Supplementary material for: Cellular Characteristics and Protein Signatures of Human Adipose Tissues from Donors With or Without Advanced Coronary Artery Disease
Source: Biomedicines. 2024 Oct 25;12(11):2453. doi: 10.3390/biomedicines12112453 (PMC11592159; doi:10.3390/biomedicines12112453)
Supplement: Supplementary file 1 [file biomedicines-12-02453-s001.zip › biomedicines-3207971-supplementary.pdf]

Supplemental Methods

Mass spectrometry and proteomics analysis. Sample preparation. Cells were solubilized in RIPA buffer and DNA sheared using a probe-tip sonicator (Branson Ultrasonic Corporation, Danbury, CT, 3 × 10 seconds) operating at 30% power and a 30% duty cycle with the samples on ice. Samples were then centrifuged (14,000 × g) at 4°C and the supernatant collected. Protein concentrations were measured relative to bovine serum albumin protein standards using the bicinchoninic acid assay (Thermo Scientific Pierce, Waltham, MA). Fifty micrograms of protein from each sample were reduced using 5 mM TCEP (tris(2-carboxyethyl) phosphine hydrochloride, Strem Chemicals, Newburyport, MA) for 20 minutes at 56°C. Samples were cooled to room temperature and alkylated for 30 minutes in the dark with 10 mM iodoacetamide (G-Biosciences, St. Louis, MO). Protein was precipitated for 2 hours at -20°C with >10-fold excess volume of ethanol. Pellets were washed twice with ice-cold ethanol, centrifuged (14,000 × g, 4°C), and resuspended in 100 mM ABC containing 1 mM $CaCl_2$ and trypsin (Sequencing grade, modified, Promega Co, Madison, WI) that was 5% the mass of protein to be digested. Incubation was carried out overnight at 37°C. Digested proteins were evaporated on a centrifugal evaporator (Savant, Thermo Fisher Scientific) and subjected to solid-phase extraction on C18 resin (Rappsilber, J., Mann, M. & Ishihama, Y. Protocol for micro-purification, enrichment, pre-fractionation and storage of peptides for proteomics using StageTips. Nat Protoc, 2, 1896–1906 (2007). Purified peptides were eluted directly into autosampler vials to be used on the LC-MS instrumentation using 100 µL elution buffer and solvent removed by vacuum centrifugation. Sample were resuspended in a volume of sample load solvent (5% formic acid (Optima grade, Thermo Fisher Scientific) and 4% acetonitrile (LC-MS-grade, Honeywell) to yield ~1 µg/µL peptides.

Chromatography / mass spectrometry. Tandem mass spectrometric analysis was performed on an Eksigent NanoLC 425 nano-UPLC System (Sciex, Framingham, MA) in direct-injection mode with a 5 µL sample loop. Fractionation was performed on a reverse-phase nano HPLC column (Acclaim PepMap 100 C18, 75 µm × 150 mm, 3 µm particle, 120 Å pore) held at 45°C with a flow rate of 350 nL/min. Solvents were blended from LC-MS-grade water and acetonitrile (Burdick & Jackson, Muskegon, MI). Mobile phase A contained 2% acetonitrile, while mobile phase B was acetonitrile. Both contained 0.1% formic acid (Optima grade, Fisher Chemical, Waltham, MA). Peptides (1 µg) were applied to the column equilibrated at 4% B and loading continued for 10 minutes. The sample loop was then removed from the flow path and the column washed for 30 seconds at starting conditions. A gradient to 38% B was executed at constant flow rate over 90 minutes followed by a 3-minute gradient to 90% B. The column was washed for 5 minutes under these conditions before being returned to starting conditions over 2 minutes. Equilibration for the next analysis was done for 10 minutes.

Analysis was performed in positive ion mode on a TripleToF 5600 quadrupole time-of-flight (QToF) mass spectrometer (Sciex, Framingham, MA). The column eluate was directed to a silica capillary emitter (SilicaTip, 20 µm ID, New Objective, Littleton, MA) maintained at 2500 V. Nitrogen nebulizer gas was held at 4 psi with the curtain gas at 23 psi. The source was kept at 150°C. Information-dependent acquisition was performed as follows: a parent ion scan was acquired over a range of 400-1250 mass units using a 250 msec accumulation time in high-sensitivity mode. This measurement was followed by MS/MS analyses of the 50 most-intense ions detected in the parent ion scan. Dynamic accumulation time was used at unit resolution and set to 45 msec with product ions detected from 100-1500 amu. Rolling collision energies were used, using manufacturer-recommended parameters. A collision energy spread of 5 was employed. SWATH analysis was performed starting with a parent ion scan using 250 msec accumulation time acquired over a range of 400-1500 mass units. SWATH MS/MS windows of variable widths, 100 in total, were generated using a variable-window calculator (Sciex). Fragmentation conditions were optimized for ions of a 2+ charge state. SWATH detection parameters were set to a mass range of m/z = 100-1500 with accumulation times of 50 msec in the high-sensitivity mode.

Proteomics data analysis. Protein identification was performed using Protein Pilot software (Sciex, Version 5.0.2) running the Paragon algorithm. Data were searched against a human proteome database containing over 20,000 manually-annotated entries in FASTA format downloaded from the Uniprot website. Searches were performed with cysteines modified (iodoacetamide). A target false-discovery rate of 0.05 and a thorough ID search effort was selected. A minimum of 95% confidence was used as a threshold for peptide identification. Relative quantification was performed using the SWATH processing microApp in the Sciex PeakView software. Peak groups were extracted with a 99% peptide confidence threshold and 1% peptide FDR limit. SWATH chromatograms were extracted in 10-minute windows with fragment ion mass tolerance set to 75 ppm. Resulting protein quantitative peak areas were further analyzed using MarkerView software (Version 1.3.1, Sciex LLC, Framingham, MA) to compare relative quantities of all detected proteins between samples. Statistical analyses including t-tests and principal component analyses, were completed for data sets using Sciex MarkerView software. Significantly different proteins were determined via t-test ($p < .05$).

Supplemental Table S1. Full demographic information for subjects with samples utilized in this study.

	Sex	Age	Race	Smoker	pack years	Height (inches)	Weight (lbs)	BMI	Pre-diabetes	diabetes	Insulin dependent	Hba1c level (5.7-6.4%)	Anti-diabetic meds	Lipid-lowering meds	Anti-hyper-tensive meds	Concomitant cardiac surgery
CABG samples																
101	Male	77	white	Past		67	211	33	No	No	No	5.6	No	Yes	Yes	aortic valve replacement
102	Male	79	white	past		68	162	24.63	No	No	No	5.6	No	Yes	Yes	
103	Male	62	white	Past	10	70	271	38.88	No	Yes	Yes	8.2	Yes	No	Yes	
104	Male	82	white	Past	40	72	210	28.5	No	Yes	No	6.9	Yes	Yes	Yes	
105	Male	75	white	No		66.26	194	30.38	No	No	No	6.5	No	Yes	Yes	
106	Male	70	white	No		69	203	29.76	No	No	No		No	Yes	No	aortic valve replacement
107	Male	66	white	No		68	235	35.73	No	Yes		9.9	Yes	Yes	No	
108	Male	72	white	past	15	64	189	32.44	No	No	No	5.9	No	Yes	Yes	
109	Female	60	white	past	15	67	162	24.7	Yes	Yes	Yes	11.3	Yes	Yes	Yes	
110	Female	78	white	No		61	204	38.7	No	No	No	5.3	No	Yes	Yes	
111	Male	68	white	No		70	244	35.01	No	Yes		9	Yes		Yes	Closure of patent foramen ovale
112	Male	59	white	past	5	68	207	31.58	No	No	No	5.7	Yes	No	Yes	MAZE and left atrial appendage ligation
113	Male	74	white	past	8	65	192	31.95	No	No	No	5.4	No	Yes	Yes	MAZE and left atrial appendage ligation
114	Male	61	white	No		71	213	29.71	No	No	No	5.1		Yes	Yes	
115	Male	75	white	past	10	68	255	34.22	No	No	No	4.7	No	Yes	No	aortic valve replacement
116	Male	63	white	No		68	179	27.31	No	No	No	5.6	No	Yes	No	aortic valve replacement
117	Male	62	white	No		70	207.7	29.4	No	No	No	5.7	No	Yes	Yes	ascending hemiarch replacement
118	Male	64	white	Past		72	278	37.7	No	No	No	5.4	No	Yes	Yes	mitral valve repair
119	Male	47	white	No		74	183.6	23.6	No	Yes	Yes	7.3	Yes	Yes	Yes	
120	Male	77	white	No		75	231	28.8	No	Yes	Yes	7.8	Yes	Yes	Yes	MAZE
121	Male	65	white	Yes		69	222.5	32.8	No	No	No	5.7	No	Yes	Yes	
122	Male	68	white	Past	5	69	147	21.8	No	No		5.3	No	Yes	No	
123	Male	66	white	Past	25	68	208	31.74	No	Yes	Yes	6.4	Yes	Yes	Yes	
124	Male	66	white	Past	25	68	199	30.2	No	Yes	Yes	6.4	Yes	Yes	Yes	
125	Male	74	white	Past	30	69.5	217	31.7	No	No		5.5	No	Yes	No	tricuspid valve repair, aortic valve replacement, left atrial appendage ligation, and

																closure of a patent foramen ovale
126	Male	58	white	No		70	200	28.8	No	Yes	No	11	Yes	No	No	
127	Male	55	white	Past		69	171	25	No	No		6	No	Yes	Yes	
128	Male	62	white	No		69	187	27.6	No	No		5.5	No	No	Yes	
129	Male	76	white			68	145	22.1	No	Yes	No	7.8	Yes	Yes	No	
130	Male	73	white			68	257	39.1	No	Yes	No	7.2	Yes	Yes	Yes	
131	Female	79	white	Past	1	65	171	28.5	No	Yes	No	6.4	Yes	No	Yes	
132	Male	81	white	No		63	148	26.2	No	No		5.7	No	Yes	Yes	
133	Male	70	white	Past	20	63	224	38.8	No	No		5.8	No	Yes	Yes	
134	Male	71	white	Past	20	70	250	35.9	Yes	No		6	No	No	Yes	left atrial appendage ligation
135	Male	78	white	Past	40	67	145	22.4	No	Yes	No	6.6	Yes	Yes	Yes	
136	Male	87	white	No		71	183	25.6	No	No		5.7	No	No	Yes	
137	Male	73	white	No		69	157	23.2	No	No		5.8	No	Yes	Yes	
138	Male	71	white	Yes		70	242	34.8	No	No		5.9	No	Yes	No	left atrial appendage ligation
VR samples																
201	Male	83	white	No		70	196	28.1	No	No	No	5.7	No	Yes	Yes	MAZE and left atrial appendage ligation
202	Female	67	white	Past	10	66	157	25.02	No	No	No	5.9	No	No	Yes	MAZE and left atrial appendage ligation
203	Male	65	white	No		72	180	24.41	No	No	No	5.2	No	No	No	
204	Male	37	white	Past	5	72	188.8	25.7	No	No	No		No	No	No	
205	Male	38	American Indian or Alaska Native	Yes	20	70	178	25.5	No	No	No	4.7	No	No	No	Closure of patent foramen ovale
206	Female	72	white	No		50	149	28.54	No	No	No		No	No	Yes	MAZE, tricuspid repair, and left atrial appendage ligation
207	Male	71	white	No		67	156	23.04	No	No	No	5.3	No	No	No	MAZE and left atrial appendage ligation
208	Male	57	white	No		73	167	22.65	No	No	No	4.8	No	No	No	MAZE and left atrial appendage ligation
209	Female	63	white	No		63	136	24.26	No	No	No	5.5	No	No	No	
210	Female	78	white	No		64	109	18.74	No	No	No	5.5	No	Yes	Yes	left atrial appendage ligation
211	Female	79	white	Past	17	62	131	24.03	No	No	No	5.5	No	Yes	Yes	Closure of patent foramen ovale and

																left atrial appendage ligation
212	Male	68	white	Past	10	67	176	27.57	No	No	No	5.4	No	Yes	Yes	MAZE
213	Male	72	white	Past	20	72	215	29.16	No	No	No	6.1	No	No	Yes	MAZE and left atrial appendage ligation
214	Male	59	white			65	234	37.61	No	No	No	5.3	No	No	Yes	MAZE and left atrial appendage ligation
215	Male	65	white	Past	40	75	372	45.28	Yes	Yes	No	6.4	Yes		Yes	MAZE, aortic valve replacement, and ascending aortic repair
216	Male	55	white	No		69	137	20.32	No	No		5.4	No	Yes	No	Closure of patent foramen ovale and left atrial appendage ligation

Supplemental Table S2. Proteins differentially expressed between CABG PVAT and CABG SubQ in SWATH #1. Accession number, name, p-value, means, fold change, and log fold change are shown for each protein with a p-value <= 0.05 and log fold change >=0.3 or <=-0.3.

Peak Name	p-value	Mean 1	Mean 2	Fold Change	Log (Fold Change)
sp|P19105|ML12A_HUMAN	0.033	1375.628	8791.998	0.156	-0.806
sp|O00764|PDXK_HUMAN	0.040	2464.079	14237.322	0.173	-0.762
sp|P02042|HBD_HUMAN	0.016	11353.682	32986.708	0.344	-0.463
sp|Q96I99|SUCB2_HUMAN	0.044	823.229	2216.892	0.371	-0.430
sp|Q13885|TBB2A_HUMAN	0.011	4114.037	10713.221	0.384	-0.416
sp|P62826|RAN_HUMAN	0.047	2500.485	6414.245	0.390	-0.409
sp|P25311|ZA2G_HUMAN	0.043	7405.053	16920.248	0.438	-0.359
sp|P0DOX2|IGA2_HUMAN	0.047	2694.139	6100.239	0.442	-0.355
sp|P62879|GBB2_HUMAN	0.007	2304.723	5150.179	0.448	-0.349
sp|P23284|PPIB_HUMAN	0.026	7007.419	15406.165	0.455	-0.342
sp|Q9UL46|PSME2_HUMAN	0.023	9400.311	4509.350	2.085	0.319
sp|Q12905|ILF2_HUMAN	0.021	38478.947	16744.678	2.298	0.361
sp|P42167|LAP2B_HUMAN	0.046	3978.799	1709.331	2.328	0.367
sp|P09493|TPM1_HUMAN	0.046	1267.038	543.367	2.332	0.368
sp|P52565|GDIR1_HUMAN	0.035	5303.163	2170.717	2.443	0.388
sp|P04233|HG2A_HUMAN	0.046	766.499	306.800	2.498	0.398
sp|Q15365|PCBP1_HUMAN	0.039	4465.413	1782.555	2.505	0.399
sp|Q92597|NDRG1_HUMAN	0.045	2092.836	818.425	2.557	0.408
sp|P60174|TPIS_HUMAN	0.030	3491.732	1322.994	2.639	0.421
sp|P52566|GDIR2_HUMAN	0.039	23953.664	9068.623	2.641	0.422
sp|Q9Y394|DHRS7_HUMAN	0.013	2724.178	1011.740	2.693	0.430
sp|P53621|COPA_HUMAN	0.029	4148.423	1536.752	2.699	0.431
sp|P11047|LAMC1_HUMAN	0.044	42127.114	15225.623	2.767	0.442
sp|Q13011|ECH1_HUMAN	0.049	3943.856	1413.096	2.791	0.446
sp|P56385|ATP5I_HUMAN	0.045	6060.873	2168.862	2.794	0.446
sp|P53396|ACLY_HUMAN	0.041	11148.583	3962.629	2.813	0.449
sp|Q96IU4|ABHEB_HUMAN	0.010	10881.382	3834.581	2.838	0.453
sp|O75396|SC22B_HUMAN	0.025	2062.438	725.797	2.842	0.454
sp|Q92522|H1X_HUMAN	0.016	6107.236	2110.327	2.894	0.461

sp|P52272|HNRPM_HUMAN	0.029	6846.919	2339.794	2.926	0.466
sp|P04271|S100B_HUMAN	0.036	2974.163	1015.009	2.930	0.467
sp|Q9Y3F4|STRAP_HUMAN	0.011	645.134	219.432	2.940	0.468
sp|Q13561|DCTN2_HUMAN	0.041	6142.837	2074.962	2.960	0.471
sp|P60866|RS20_HUMAN	0.031	25921.997	8719.356	2.973	0.473
sp|P0DP25|CALM3_HUMAN	0.013	8379.500	2806.811	2.985	0.475
sp|P07355|ANXA2_HUMAN	0.042	2803.359	929.938	3.015	0.479
sp|Q07507|DERM_HUMAN	0.019	6941.590	2276.091	3.050	0.484
sp|Q15661|TRYB1_HUMAN	0.018	756.494	241.435	3.133	0.496
sp|Q14847|LASP1_HUMAN	0.047	9786.864	3106.374	3.151	0.498
sp|P0DMV9|HS71B_HUMAN	0.021	24109.013	7533.000	3.200	0.505
sp|Q86YZ3|HORN_HUMAN	0.016	10035.746	3133.461	3.203	0.506
sp|Q14195|DPYL3_HUMAN	0.027	4785.377	1483.138	3.227	0.509
sp|P01859|IGHG2_HUMAN	0.029	17914.910	5527.095	3.241	0.511
sp|P02730|B3AT_HUMAN	0.029	5829.306	1690.668	3.448	0.538
sp|O15511|ARPC5_HUMAN	0.050	3604.310	1040.149	3.465	0.540
sp|P49773|HINT1_HUMAN	0.010	2582.690	742.380	3.479	0.541
sp|P48444|COPD_HUMAN	0.041	1505.242	428.168	3.516	0.546
sp|O15144|ARPC2_HUMAN	0.028	4024.947	1143.201	3.521	0.547
sp|P26641|EF1G_HUMAN	0.029	4187.991	1186.455	3.530	0.548
sp|P08311|CATG_HUMAN	0.015	12254.053	3424.559	3.578	0.554
sp|P49368|TCPG_HUMAN	0.013	13619.977	3788.940	3.595	0.556
sp|P18206|VINC_HUMAN	0.037	3211.248	892.891	3.596	0.556
sp|Q13423|NNTM_HUMAN	0.042	2260.173	591.692	3.820	0.582
sp|P49327|FAS_HUMAN	0.019	16897.469	4350.858	3.884	0.589
sp|P09429|HMGB1_HUMAN	0.006	4361.003	1122.196	3.886	0.590
sp|Q9UHD8|SEPT9_HUMAN	0.001	19377.955	4923.799	3.936	0.595
sp|P20700|LMNB1_HUMAN	0.035	9160.156	2319.400	3.949	0.597
sp|P62805|H4_HUMAN	0.045	11893.091	2983.222	3.987	0.601
sp|P53990|IST1_HUMAN	0.038	18830.658	4504.295	4.181	0.621
sp|Q13838|DX39B_HUMAN	0.009	3425.240	818.545	4.185	0.622
sp|P51610|HCFC1_HUMAN	0.050	6776.917	1603.502	4.226	0.626
sp|P24752|THIL_HUMAN	0.036	10599.916	2485.026	4.266	0.630
sp|P00390|GSHR_HUMAN	0.024	13003.211	3018.448	4.308	0.634

sp|P07900|HS90A_HUMAN	0.018	63480.776	14100.639	4.502	0.653
sp|P00352|AL1A1_HUMAN	0.005	24370.028	5257.622	4.635	0.666
sp|P05090|APOD_HUMAN	0.021	3799.322	776.223	4.895	0.690
sp|P37840|SYUA_HUMAN	0.032	2923.084	595.588	4.908	0.691
sp|Q16666|IF16_HUMAN	0.038	542.719	108.914	4.983	0.697
sp|P46940|IQGA1_HUMAN	0.031	46709.879	9369.065	4.986	0.698
sp|P56134|ATPK_HUMAN	0.002	4768.667	956.428	4.986	0.698
sp|Q9UJU6|DBNL_HUMAN	0.026	11746.150	2210.760	5.313	0.725
sp|P01011|AACT_HUMAN	0.021	59039.763	10886.233	5.423	0.734
sp|Q92506|DHB8_HUMAN	0.022	2444.219	446.585	5.473	0.738
sp|P29966|MARCS_HUMAN	0.036	814.985	147.603	5.521	0.742
sp|P53597|SUCA_HUMAN	0.013	3640.485	562.401	6.473	0.811
sp|P09874|PARP1_HUMAN	0.043	40440.682	6002.521	6.737	0.828
sp|Q08211|DHX9_HUMAN	0.032	4759.566	645.909	7.369	0.867
sp|Q9UI08|EVL_HUMAN	0.026	20224.034	2593.239	7.799	0.892
sp|P04632|CPNS1_HUMAN	0.036	5274.954	640.156	8.240	0.916
sp|Q1KMD3|HNRL2_HUMAN	0.005	33942.710	3635.454	9.337	0.970
sp|O60240|PLIN1_HUMAN	0.009	20182.044	1744.075	11.572	1.063
sp|P47914|RL29_HUMAN	0.007	4986.535	275.670	18.089	1.257

Supplemental Table S3. Proteins differentially expressed between VR PVAT and VR SubQ in SWATH #1. Accession number, name, p-value, means, fold change, and log fold change are shown for each protein with a p-value <= 0.05 and log fold change >=0.3 or <=-0.3.

Peak Name	p-value	Mean 1	Mean 2	Fold Change	Log (Fold Change)
sp|P80511|S10AC_HUMAN	0.045	839.595	6919.358	0.121	-0.916
sp|Q9H299|SH3L3_HUMAN	0.012	2367.093	12782.937	0.185	-0.732
sp|P02763|A1AG1_HUMAN	0.037	5450.041	15209.344	0.358	-0.446
sp|Q14697|GANAB_HUMAN	0.019	10516.735	4333.180	2.427	0.385
sp|P01024|CO3_HUMAN	0.017	22333.819	9113.179	2.451	0.389
sp|P61019|RAB2A_HUMAN	0.050	6177.730	2485.926	2.485	0.395
sp|P15311|EZRI_HUMAN	0.047	7694.194	2907.101	2.647	0.423
sp|P62280|RS11_HUMAN	0.027	10649.290	3956.089	2.692	0.430
sp|P62851|RS25_HUMAN	0.047	4830.634	1717.996	2.812	0.449
sp|P49368|TCPG_HUMAN	0.036	5673.033	1986.641	2.856	0.456
sp|Q03252|LMNB2_HUMAN	0.041	14073.851	4636.895	3.035	0.482
sp|O00299|CLIC1_HUMAN	0.049	11524.269	3765.007	3.061	0.486
sp|P17096|HMGA1_HUMAN	0.040	2880.795	915.640	3.146	0.498
sp|P37802|TAGL2_HUMAN	0.023	12949.096	4067.911	3.183	0.503
sp|Q9Y3I0|RTCB_HUMAN	0.048	3000.375	904.075	3.319	0.521
sp|Q9NUV9|GIMA4_HUMAN	0.019	8181.555	2380.055	3.438	0.536
sp|P80748|LV321_HUMAN	0.048	3971.232	1148.016	3.459	0.539
sp|Q13162|PRDX4_HUMAN	0.046	4271.666	1229.150	3.475	0.541
sp|P08238|HS90B_HUMAN	0.042	12980.874	3563.413	3.643	0.561
sp|P19338|NUCL_HUMAN	0.039	11557.791	3156.029	3.662	0.564
sp|P09417|DHPR_HUMAN	0.015	10890.954	2902.815	3.752	0.574
sp|P06744|G6PI_HUMAN	0.017	1516.394	403.165	3.761	0.575
sp|P02462|CO4A1_HUMAN	0.019	7231.317	1908.258	3.789	0.579
sp|O75964|ATP5L_HUMAN	0.011	6665.626	1751.604	3.805	0.580
sp|P30041|PRDX6_HUMAN	0.015	10040.003	2613.615	3.841	0.584
sp|P07741|APT_HUMAN	0.016	3502.288	852.868	4.106	0.613
sp|P01011|AACT_HUMAN	0.023	18292.578	4388.112	4.169	0.620
sp|P08240|SRPRA_HUMAN	0.042	2207.957	482.427	4.577	0.661
sp|P00491|PNPH_HUMAN	0.007	9486.745	2046.479	4.636	0.666
sp|O15145|ARPC3_HUMAN	0.001	12425.121	2564.912	4.844	0.685

sp|P46777|RL5_HUMAN	0.047	28837.979	5748.126	5.017	0.700
sp|Q9Y277|VDAC3_HUMAN	0.031	2913.600	575.633	5.062	0.704
sp|P36957|ODO2_HUMAN	0.028	11582.681	2204.515	5.254	0.720
sp|P05155|IC1_HUMAN	0.023	7724.722	1436.938	5.376	0.730
sp|Q05469|LIPS_HUMAN	0.006	3176.786	510.641	6.221	0.794
sp|A0A0B4J1V0|HV315_HUMAN	0.009	12390.326	1671.496	7.413	0.870

Supplemental Table S4. Proteins differentially expressed between CABG PVAT vs VR PVAT. Show are changes with a p-value <= 0.05 and log fold change >=0.3 or <=-0.3.

Peak Name	p-value	Mean 1 CABG	Mean 2 VR	Fold Change	Log (Fold Change)
sp|Q1KMD3|HNRL2_HUMAN	0.050	1288.490	3484.010	0.370	-0.432
sp|Q15746|MYLK_HUMAN	0.006	11853.237	5448.733	2.175	0.338
sp|Q9UHD8|SEPT9_HUMAN	0.010	4059.442	1856.613	2.186	0.340
sp|P07858|CATB_HUMAN	0.022	24882.554	11292.431	2.203	0.343
sp|P49368|TCPG_HUMAN	0.019	1706.696	774.511	2.204	0.343
sp|P20930|FILA_HUMAN	0.032	8634.702	3884.765	2.223	0.347
sp|P80511|S10AC_HUMAN	0.015	934.967	403.526	2.317	0.365
sp|Q7KZF4|SND1_HUMAN	0.017	5240.143	2174.603	2.410	0.382
sp|Q92597|NDRG1_HUMAN	0.020	10348.628	4259.059	2.430	0.386
sp|P55084|ECHB_HUMAN	0.025	16077.189	6611.528	2.432	0.386
sp|P55083|MFAP4_HUMAN	0.015	51412.590	20907.231	2.459	0.391
sp|Q15661|TRYB1_HUMAN	0.016	2567.610	1037.110	2.476	0.394
sp|Q8WYJ6|SEPT1_HUMAN	0.017	21759.315	8769.910	2.481	0.395
sp|Q15417|CNN3_HUMAN	0.018	5476.385	2202.624	2.486	0.396
sp|P56134|ATPK_HUMAN	0.021	13369.826	5287.234	2.529	0.403
sp|P62280|RS11_HUMAN	0.010	7143.556	2815.115	2.538	0.404
sp|P52272|HNRPM_HUMAN	0.031	5935.245	2312.969	2.566	0.409
sp|P06737|PYGL_HUMAN	0.048	15154.807	5890.085	2.573	0.410
sp|P50213|IDH3A_HUMAN	0.008	2143.135	826.157	2.594	0.414
sp|P29966|MARCS_HUMAN	0.005	8384.699	3176.717	2.639	0.422
sp|P05109|S10A8_HUMAN	0.010	11833.815	4371.714	2.707	0.432
sp|P78527|PRKDC_HUMAN	0.002	43401.378	16008.451	2.711	0.433
sp|Q07666|KHDR1_HUMAN	0.010	27850.180	9981.327	2.790	0.446
sp|P30049|ATPD_HUMAN	0.009	33123.736	11854.619	2.794	0.446
sp|P04271|S100B_HUMAN	0.020	36938.047	12891.886	2.865	0.457
sp|P15088|CBPA3_HUMAN	0.009	17859.042	6163.675	2.897	0.462
sp|P06703|S10A6_HUMAN	0.036	22713.100	7831.808	2.900	0.462
sp|Q08211|DHX9_HUMAN	0.009	7269.966	2498.097	2.910	0.464
sp|P04275|VWF_HUMAN	0.003	12388.083	4254.373	2.912	0.464
sp|Q12905|ILF2_HUMAN	0.033	21086.124	7139.887	2.953	0.470
sp|Q14847|LASP1_HUMAN	0.030	9225.941	3117.441	2.959	0.471

sp|Q01469|FABP5_HUMAN	0.002	22758.406	7608.921	2.991	0.476
sp|P00390|GSHR_HUMAN	0.006	5223.741	1740.603	3.001	0.477
sp|Q96AG4|LRC59_HUMAN	0.024	12571.111	4185.286	3.004	0.478
sp|P0DMV9|HS71B_HUMAN	0.002	4306.342	1413.175	3.047	0.484
sp|Q15046|SYK_HUMAN	0.0002	2088.367	680.215	3.070	0.487
sp|P49411|EFTU_HUMAN	0.009	784.003	236.616	3.313	0.520
sp|P12956|XRCC6_HUMAN	0.013	727.365	216.438	3.361	0.526
sp|P51888|PRELP_HUMAN	0.038	2815.272	837.384	3.362	0.527
sp|Q02952|AKA12_HUMAN	0.005	45455.640	13423.135	3.386	0.530
sp|P61586|RHOA_HUMAN	0.011	12777.352	3685.589	3.467	0.540
sp|Q13561|DCTN2_HUMAN	0.008	19442.582	5196.374	3.742	0.573
sp|A0A0C4DH29|HV103_HUMAN	0.029	1902.995	500.400	3.803	0.580
sp|P28482|MK01_HUMAN	0.021	8620.454	2217.349	3.888	0.590
sp|Q9UI08|EVL_HUMAN	0.0005	3386.872	865.534	3.913	0.593
sp|Q9P2R7|SUCB1_HUMAN	0.0004	70429.514	16305.913	4.319	0.635
sp|Q9UJ70|NAGK_HUMAN	0.009	8065.553	1727.119	4.670	0.669
sp|P42167|LAP2B_HUMAN	0.008	4268.259	889.178	4.800	0.681
sp|P27816|MAP4_HUMAN	0.008	529.860	102.258	5.182	0.714
sp|O43399|TPD54_HUMAN	0.002	17326.079	3252.882	5.326	0.726
sp|P35998|PRS7_HUMAN	0.002	49897.344	9196.160	5.426	0.734
sp|P08575|PTPRC_HUMAN	0.000	6045.151	1046.668	5.776	0.762
sp|Q9Y230|RUVB2_HUMAN	0.029	46874.685	7701.063	6.087	0.784
sp|P81605|DCD_HUMAN	0.001	5315.219	792.548	6.706	0.826
sp|Q14152|EIF3A_HUMAN	0.00000005	36628.072	4492.329	8.153	0.911
sp|P49753|ACOT2_HUMAN	0.00001	873.150	100.843	8.659	0.937
sp|Q00610|CLH1_HUMAN	0.013	1165.850	129.338	9.014	0.955
sp|P19105|ML12A_HUMAN	0.0001	5717.894	277.753	20.586	1.314

Supplemental Table S5. Proteins differentially expressed between CABG SubQ vs VR SubQ. Shown are comparisons with a p-value <= 0.05 and log fold change >=0.3 or <=-0.3.

Peak Name	p-value	Mean 1 CABG	Mean 2 VR	Fold Change	Log (Fold Change)
sp|Q16363|LAMA4_HUMAN	0.0003	7531.355	638.096	11.803	1.072
sp|P62310|LSM3_HUMAN	0.002	31179.543	4065.257	7.670	0.885
sp|P60660|MYL6_HUMAN	0.002	11326.049	1598.324	7.086	0.850
sp|Q9NR31|SAR1A_HUMAN	0.003	3737.269	529.355	7.060	0.849
sp|Q9BR76|COR1B_HUMAN	0.003	9821.400	820.256	11.974	1.078
sp|P62851|RS25_HUMAN	0.005	13058.428	1899.513	6.875	0.837
sp|P05388|RLA0_HUMAN	0.006	8133.285	2084.924	3.901	0.591
sp|P62913|RL11_HUMAN	0.008	22226.796	7361.562	3.019	0.480
sp|P30041|PRDX6_HUMAN	0.008	11177.131	2723.491	4.104	0.613
sp|P16885|PLCG2_HUMAN	0.008	7409.491	1003.411	7.384	0.868
sp|P17987|TCPA_HUMAN	0.010	87469.705	11705.116	7.473	0.873
sp|P07814|SYEP_HUMAN	0.011	1960.839	393.146	4.988	0.698
sp|P55083|MFAP4_HUMAN	0.011	4872.118	1496.332	3.256	0.513
sp|Q96AC1|FERM2_HUMAN	0.012	2021.512	955.605	2.115	0.325
sp|P50453|SPB9_HUMAN	0.013	7834.598	1493.138	5.247	0.720
sp|P05155|IC1_HUMAN	0.017	10793.895	1614.220	6.687	0.825
sp|P08240|SRPRA_HUMAN	0.017	2723.377	544.245	5.004	0.699
sp|P63173|RL38_HUMAN	0.018	8940.967	2750.044	3.251	0.512
sp|P16157|ANK1_HUMAN	0.018	2288.258	515.872	4.436	0.647
sp|O15533|TPSN_HUMAN	0.018	2464.481	550.605	4.476	0.651
sp|Q86YZ3|HORN_HUMAN	0.018	4148.906	951.479	4.360	0.640
sp|P08758|ANXA5_HUMAN	0.020	15840.223	3217.583	4.923	0.692
sp|P62249|RS16_HUMAN	0.021	94581.399	3392.749	27.878	1.445
sp|O14735|CDIPT_HUMAN	0.021	12917.420	2927.710	4.412	0.645
sp|P00491|PNPH_HUMAN	0.022	12058.388	1746.369	6.905	0.839
sp|Q15847|ADIRF_HUMAN	0.023	3625.798	775.348	4.676	0.670
sp|P27797|CALR_HUMAN	0.024	8125.456	1391.819	5.838	0.766
sp|Q13151|ROA0_HUMAN	0.025	3591.918	1260.710	2.849	0.455
sp|P61353|RL27_HUMAN	0.025	15572.148	2888.296	5.391	0.732
sp|Q9H3K6|BOLA2_HUMAN	0.025	3504.978	786.172	4.458	0.649
sp|P62917|RL8_HUMAN	0.026	5655.504	1381.096	4.095	0.612

sp|P49591|SYSC_HUMAN	0.026	2408.333	587.536	4.099	0.613
sp|O60506|HNRPQ_HUMAN	0.028	4430.359	1129.573	3.922	0.594
sp|P22087|FBRL_HUMAN	0.028	11301.209	4081.307	2.769	0.442
sp|P29590|PML_HUMAN	0.030	7771.716	1830.521	4.246	0.628
sp|Q15121|PEA15_HUMAN	0.032	3218.075	243.908	13.194	1.120
sp|P04632|CPNS1_HUMAN	0.034	818.387	185.144	4.420	0.645
sp|O75964|ATP5L_HUMAN	0.034	6423.407	1683.968	3.814	0.581
sp|Q13361|MFAP5_HUMAN	0.036	30447.485	6527.717	4.664	0.669
sp|Q9HC38|GLOD4_HUMAN	0.038	1144.403	274.321	4.172	0.620
sp|P22061|PIMT_HUMAN	0.038	3980.124	1080.924	3.682	0.566
sp|P35232|PHB_HUMAN	0.040	51745.446	13110.688	3.947	0.596
sp|Q14112|NID2_HUMAN	0.040	2782.038	723.442	3.846	0.585
sp|Q86UP2|KTN1_HUMAN	0.041	7189.355	2422.999	2.967	0.472
sp|Q92688|AN32B_HUMAN	0.041	11561.550	2415.324	4.787	0.680
sp|P19827|ITIH1_HUMAN	0.045	1740.170	454.536	3.828	0.583
sp|P01859|IGHG2_HUMAN	0.045	7522.052	2558.441	2.940	0.468
sp|P31939|PUR9_HUMAN	0.047	11705.010	3286.724	3.561	0.552
sp|P63165|SUMO1_HUMAN	0.048	5139.490	1459.520	3.521	0.547
sp|P09496|CLCA_HUMAN	0.049	3330.262	964.084	3.454	0.538
sp|Q13228|SBP1_HUMAN	0.049	3922.596	1056.206	3.714	0.570
sp|Q13162|PRDX4_HUMAN	0.049	7350.440	1356.019	5.421	0.734
sp|P84090|ERH_HUMAN	0.050	12176.442	1366.624	8.910	0.950
sp|P15311|EZRI_HUMAN	0.050	12282.095	3274.514	3.751	0.574
sp|P19367|HXK1_HUMAN	0.050	14563.659	4632.711	3.144	0.497

Supplemental Table S6. Proteins differentially expressed between CABG PVAT non-diabetic and CABG PVAT diabetic with a p-value <= 0.05 and log fold change >=0.3 or <=-0.3.

Peak Name	p-value	Mean 1 CABG	Mean 2 VR	Fold Change	Log (Fold Change)
sp|P41567|EIF1_HUMAN	0.009	6836.215	2698.219	2.534	0.404
sp|O75663|TIPRL_HUMAN	0.019	20521.017	8185.911	2.507	0.399
sp|Q9UDW1|QCR9_HUMAN	0.022	125205.960	254899.774	0.491	-0.309
sp|P03952|KLKB1_HUMAN	0.024	56295.295	22077.031	2.550	0.407
sp|Q08AF3|SLFN5_HUMAN	0.025	5911.523	2921.005	2.024	0.306
sp|P25685|DNJB1_HUMAN	0.025	33532.203	8627.581	3.887	0.590
sp|P79483|DRB3_HUMAN	0.027	82707.486	40152.466	2.060	0.314
sp|P07858|CATB_HUMAN	0.029	94569.664	32334.628	2.925	0.466
sp|Q04637|IF4G1_HUMAN	0.037	25794.370	7147.495	3.609	0.557
sp|P69892|HBG2_HUMAN	0.039	731940.512	117433.050	6.233	0.795
sp|P48506|GSH1_HUMAN	0.049	60900.126	17732.601	3.434	0.536
